# Chemical Synthesis and Structural Determination of the Inositol Glycan Head of Plant Sphingolipid GIPC in Brassicaceae

**DOI:** 10.1002/chem.202501987

**Published:** 2025-07-25

**Authors:** Yuta Umemura, Majidul Islam, Rumana Yesmin Hasi, Toshiki Ishikawa, Minoru Nagano, Takuji Oka, Naoko Komura, Akihiro Imamura, Hideharu Ishida, Hiromune Ando, Tamotsu Tanaka, Hide‐Nori Tanaka

**Affiliations:** ^1^ The United Graduate School of Agricultural Science Gifu University 1‐1 Yanagido Gifu‐shi Gifu 501–1193 Japan; ^2^ Graduate School of Technology, Industrial and Social Sciences Tokushima University 2‐1 Minami‐Josanjima‐cho Tokushima‐shi Tokushima 770–8513 Japan; ^3^ Graduate School of Science and Engineering Saitama University 255 Shimo‐Okubo Saitama‐shi Saitama 338–8570 Japan; ^4^ College of Life Sciences Ritsumeikan University 1‐1‐1 Noji‐higashi Kusatsu‐shi Shiga 525–8577 Japan; ^5^ Department of Biotechnology and Life Sciences Sojo University 4‐22‐1 Ikeda Kumamoto‐shi Kumamoto 860‐0082 Japan; ^6^ Institute for Glyco‐core Research (iGCORE) Gifu University 1‐1 Yanagido Gifu‐shi Gifu 501–1193 Japan; ^7^ Department of Applied Bioorganic Chemistry Gifu University 1‐1 Yanagido Gifu‐shi Gifu 501–1193 Japan

**Keywords:** chemical synthesis, glycosylinositol phosphoceramide, inositol glycan, plant sphingolipid, structural determination

## Abstract

Glycosylinositol phosphoceramides (GIPCs), a major class of plant sphingolipids, are key components of the plasma membrane and play important roles in plants. These molecules consist of a ceramide 1‐phosphate tail attached to an inositol glycan (IG) head, which typically contains an d‐glucuronic acid (GlcA)‐α(1→2)‐*myo*‐inositol structure. The second monosaccharide linked to the O‐4 position of the GlcA residue differs depending on plant species and cell tissues. In *Arabidopsis*, the predominant second monosaccharide has been identified as hexose (Hex) using LC‐MS/MS analysis. However, the stereochemistry and anomeric configuration of the Hex have not yet been determined using spectroscopic techniques. In this study, we chemically synthesized three different Hex‐type IGs in which the GlcA residue binds to d‐glucose, d‐galactose, or d‐mannose (Man) as the second monosaccharide. Using the synthetic standards, we determined the IG head structure of naturally occurring GIPCs isolated from cabbage by using several analytical methods. We successfully confirmed that Hex as the second monosaccharide linked to the GlcA residue is d‐Man with an α‐glycosidic bond.

## Graphical abstract

The first experimental identification of the IG head structure of naturally occurring plant GIPCs in Brassicaceae was achieved through the chemical synthesis of three hexose‐type IGs containing d‐glucose, d‐galactose, or d‐mannose as the second monosaccharide linked to d‐glucuronic acid‐α(1→2)‐myo‐inositol. Comparison with synthetic standards using analytical techniques revealed that natural IG from cabbage contains d‐mannose with an α‐glycosidic linkage as the second monosaccharide.

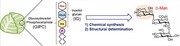

## 1 Introduction

**Figure 1:**
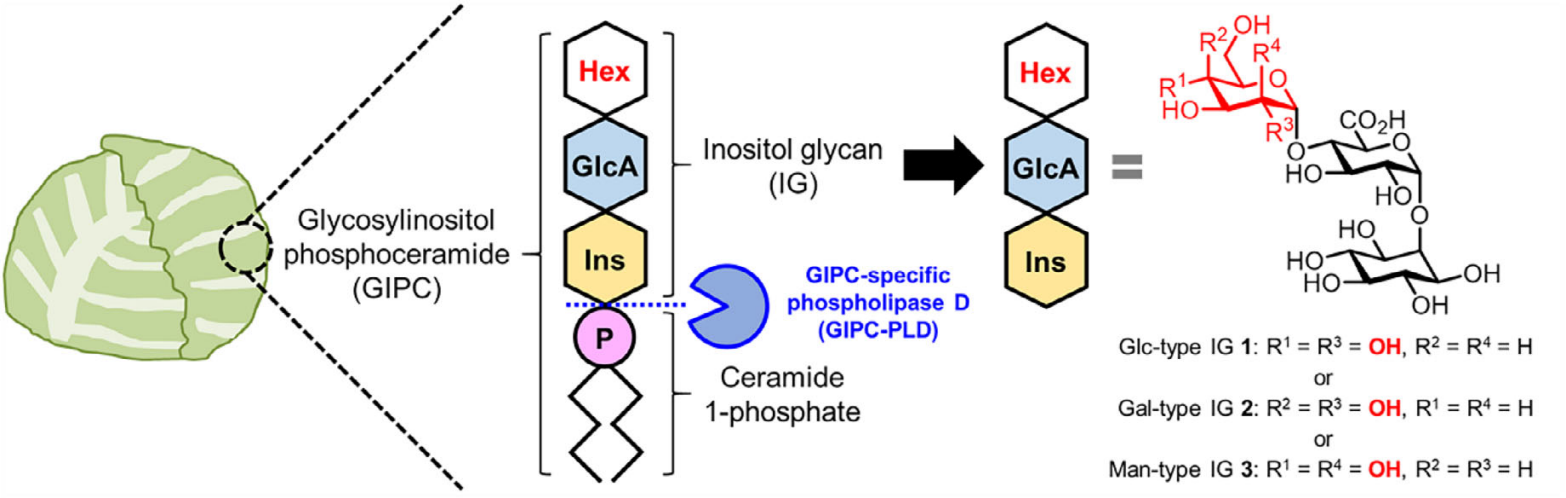
Schematic representation of a GIPC structure in Brassicaceae and plausible chemical structures of the IG head: Glc‐type IG **1**, Gal‐type IG **2**, and Man‐type IG **3**.

Glycosylinositol phosphoceramides (GIPCs), which comprise a ceramide 1‐phosphate tail linked to an inositol glycan (IG) head, are a major class of plant sphingolipids and the main components of the plasma membrane.^[^ ^1^, ^2^ ^]^ They are present on the outer leaflet of the plasma membrane and play important roles in plants. For instance, previous studies have demonstrated that GIPCs behave as lipid raft‐like nanodomain molecules, such as mammalian gangliosides,^[^ ^3^, ^4^ ^]^ and serve as a receptor for plant pathogen toxin from *Phytophthora infestans* ^[^ ^5^ ^]^ and a salt sensor for the Ca^2+^ influx channel.^[^ ^6^ ^]^ The IG head of GIPCs has a common structure of d‐glucuronic acid (GlcA)‐α(1→2)‐*myo*‐inositol (Ins) (Figure 1).^[^ ^7^ ^]^ The second monosaccharide linked to the O‐4 position of the GlcA residue differs depending on the plant species and cell tissues, and has been identified as hexose (Hex), hexosamine (HexN), and *N*‐acetylhexosamine (HexNAc) by LC‐MS/MS analysis.^[^ ^8^, ^9^, ^10^ ^]^ In *Arabidopsis*, Hex is most commonly found as the second monosaccharide.^[^ ^11^, ^12^ ^]^ Previously, Mortimer and coworkers genetically identified GIPC mannosyltransferase GMT1 belonged to glycosyltransferase family 64 as an enzyme that transfers d‐mannose (Man) from GDP‐d‐Man to the GlcA‐Ins moiety of GIPC with α‐glycosidic bond formation.^[^ ^13^ ^]^ A disaccharyl inositol with the d‐Man‐α(1→4)‐d‐GlcA‐α(1→2)‐*myo*‐Ins sequence has been isolated from cultured rose cells.^[^ ^14^ ^]^ Although Hex may be d‐Man, no molecular‐level experiments, such as in vitro production of Hex‐type GIPC or IG using recombinant GMT1, have been performed. In addition, purification of naturally occurring GIPCs remains challenging because of the difficulty of concomitant separation. To date, the stereochemistry and anomeric configuration of Hex have not been determined using spectroscopic methods. Herein, we report chemical synthesis and structural determination of the Hex‐type IG head of GIPC in Brassicaceae. Three different Hex‐type IGs **1**–**3**, in which the GlcA residue binds to d‐glucose (Glc), d‐galactose (Gal), or d‐Man as the second monosaccharide, were chemically synthesized. A naturally occurring IG was obtained by enzymatic degradation of GIPCs isolated from cabbage using GIPC‐specific phospholipase D (GIPC‐PLD).^[^ ^15^, ^16^, ^17^, ^18^, ^19^, ^20^ ^]^ Using synthetic standards, the structure of the natural IG was determined by LC‐MS/MS, monosaccharide composition, and ^1^H NMR analyses.

**Scheme 1:**
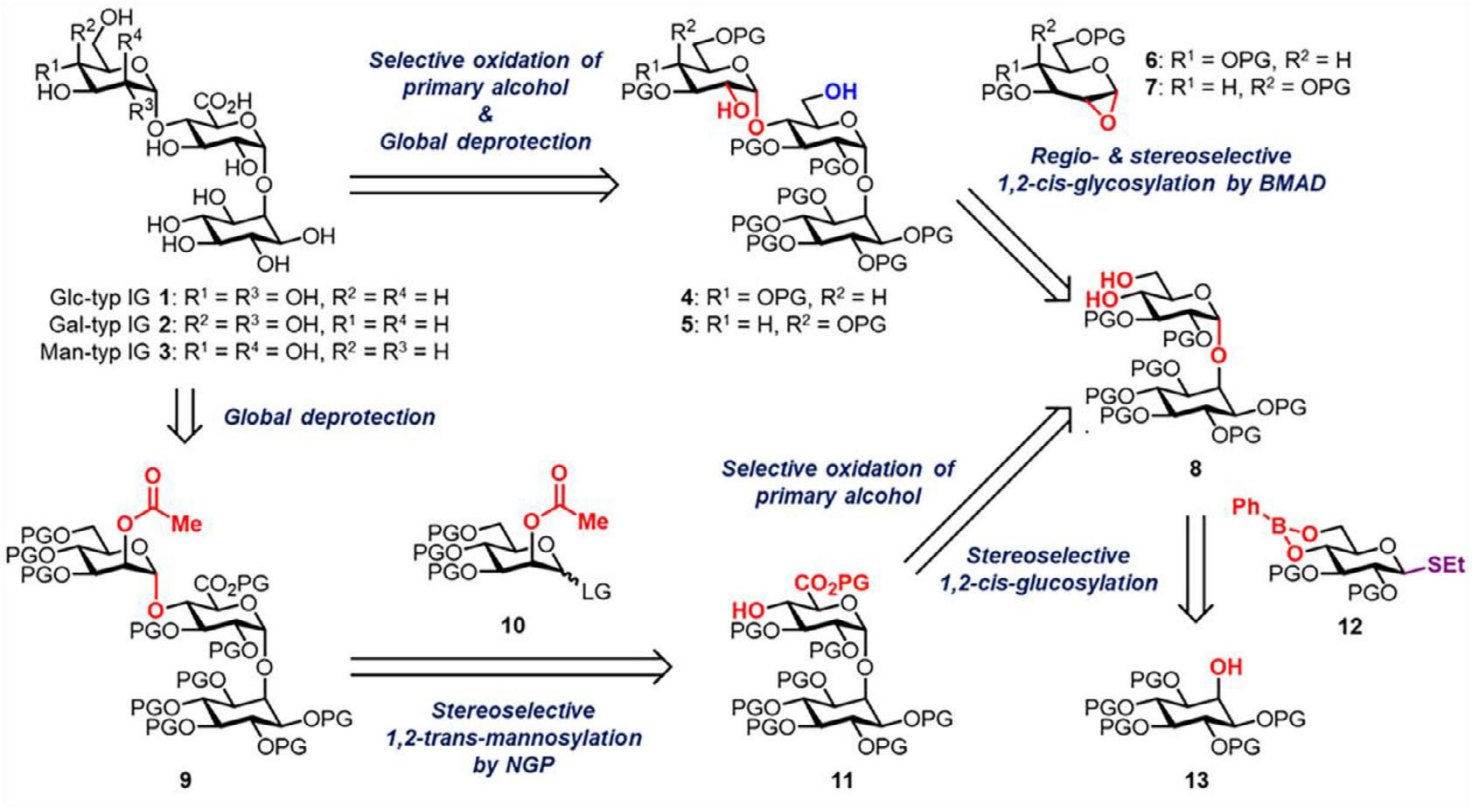
Retrosynthetic analysis of IGs **1**–**3**. BMAD: Boron‐mediated aglycon delivery; LG: Leaving group; NGP: Neighboring group participation; PG: Protecting group.

As illustrated in Scheme 1, we performed retrosynthetic analysis of IGs **1**–**3**. The target molecules possess synthetically challenging α‐glycosidic linkages. Prior to TEMPO‐mediated selective primary alcohol oxidation^[^ ^21^ ^]^ of **4** or **5**, the non‐reducing terminal α‐(1→4)‐glycosidic linkage in the framework of Glc/Gal‐type IGs **1** and **2** was proposed to originate from regio‐ and stereoselective 1,2‐*cis*‐glycosylation of 4,6‐diol acceptor **8** with 1,2‐anhydro donor **6** or **7** via boron‐mediated aglycon delivery (BMAD), which was developed by the Takahashi and Toshima group.^[^ ^22^, ^23^ ^]^ For the α‐glycosidic bond formation of Man‐type IG **3**, 1,2‐*trans*‐glycosylation by conventional neighboring group participation between 2‐*O*‐acetyl donor **10** and 4‐mono‐ol acceptor **11**, which may be converted from **8**, was envisioned. The pseudodisaccharide framework of **8** may be formed through coupling of Glc thioglycoside donor **12** using Ins acceptor **13** by 4,6‐*O*‐phenylboronic ester protecting group‐directed 1,2‐*cis*‐α‐glucosylation previously reported by the Crich group.^[^ ^24^ ^]^ The use of the cyclic boronic ester protecting group offers not only stereocontrol in glucosylation, but also easy and quantitative removal by phase‐switching aqueous workup using a 1.0 m aqueous solution of sorbitol and sodium carbonate,^[^ ^25^ ^]^ thus affording the corresponding 4,6‐diol **8**.

## 2 Results and Discussion

**Scheme 2:**
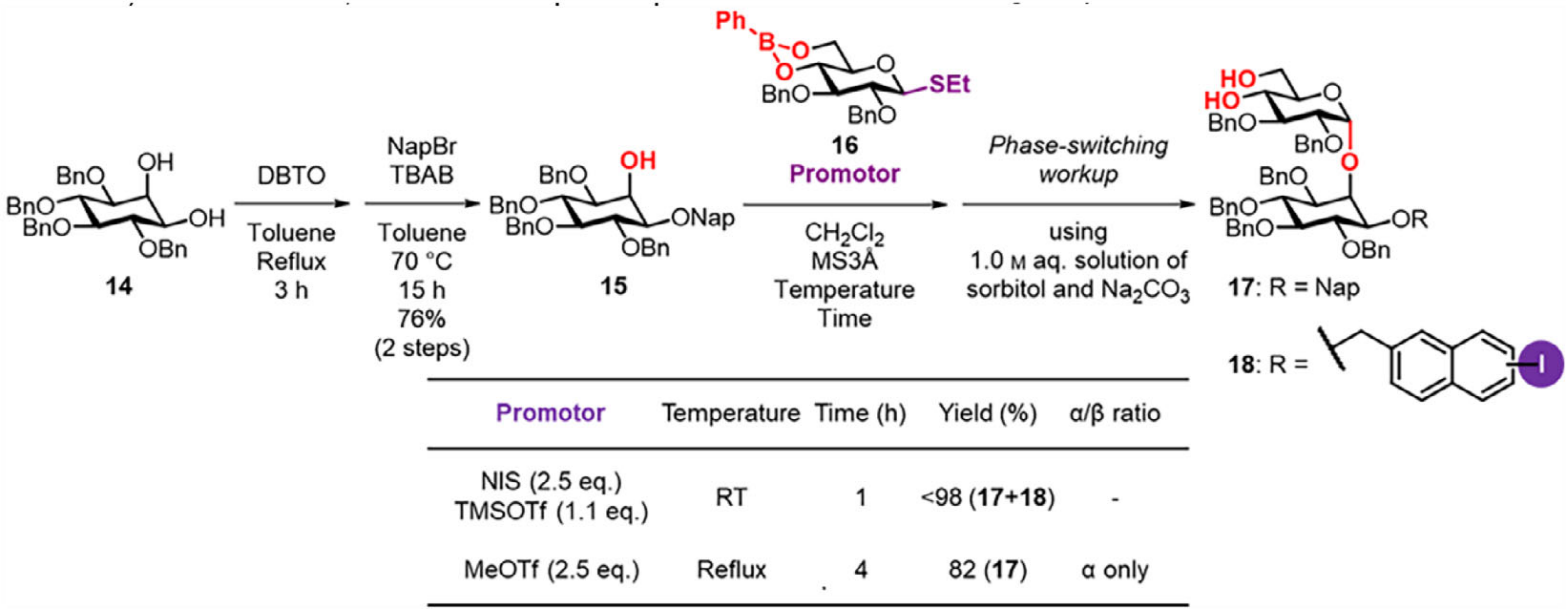
Preparation of glucosyl inositol **17** as the common intermediate in the synthesis of IGs **1**–**3**. DBTO: dibutyltin oxide; NapBr: 2‐naphthylmethyl bromide; TBAB: tetrabutylammonium bromide; NIS: *N*‐iodosuccinimide; TMSOTf: trimethylsilyl trifluoromethanesulfonate; MeOTf: methyl trifluoromethanesulfonate.

The synthesis commenced with the preparation of glucosyl inositol **17**, which served as a common intermediate for the synthesis of IGs **1**–**3** (Scheme 2). Dibutyltin oxide‐mediated regioselective *O*‐alkylation with NapBr of a known 1,2‐*cis*‐diol **14** ^[^ ^26^ ^]^ afforded the corresponding 2‐mono‐ol **15** in 76% yield. The 4,6‐*O*‐phenylboronate ester protecting group‐directed α‐glucosylation of Ins acceptor **15** with thioglycoside Glc donor **16** was performed at room temperature using a reported promotor system (NIS‐TMSOTf).^[^ ^16^ ^]^ However, the subsequent phase‐switching aqueous workup yielded a mixture of the desired product **17** and an iodine adduct **18**. This undesirable outcome was attributed to the generation of highly reactive iodonium cation in the presence of electron‐rich Nap group. To suppress the iodine addition side‐reaction, we changed to an alternative promoter system using MeOTf for **16**. Upon increasing the reaction temperature to reflux, the glucosylation proceeded smoothly. Subsequent aqueous workup afforded the pseudodisaccharide **17** in 82% yield with complete α‐selectivity. This result is consistent with our recent study demonstrating that the MeOTf‐promoted glucosylation of sterically hindered and electron‐rich alcohols using **16** proceeds in an α‐selective manner.^[^ ^27^ ^]^

**Scheme 3:**
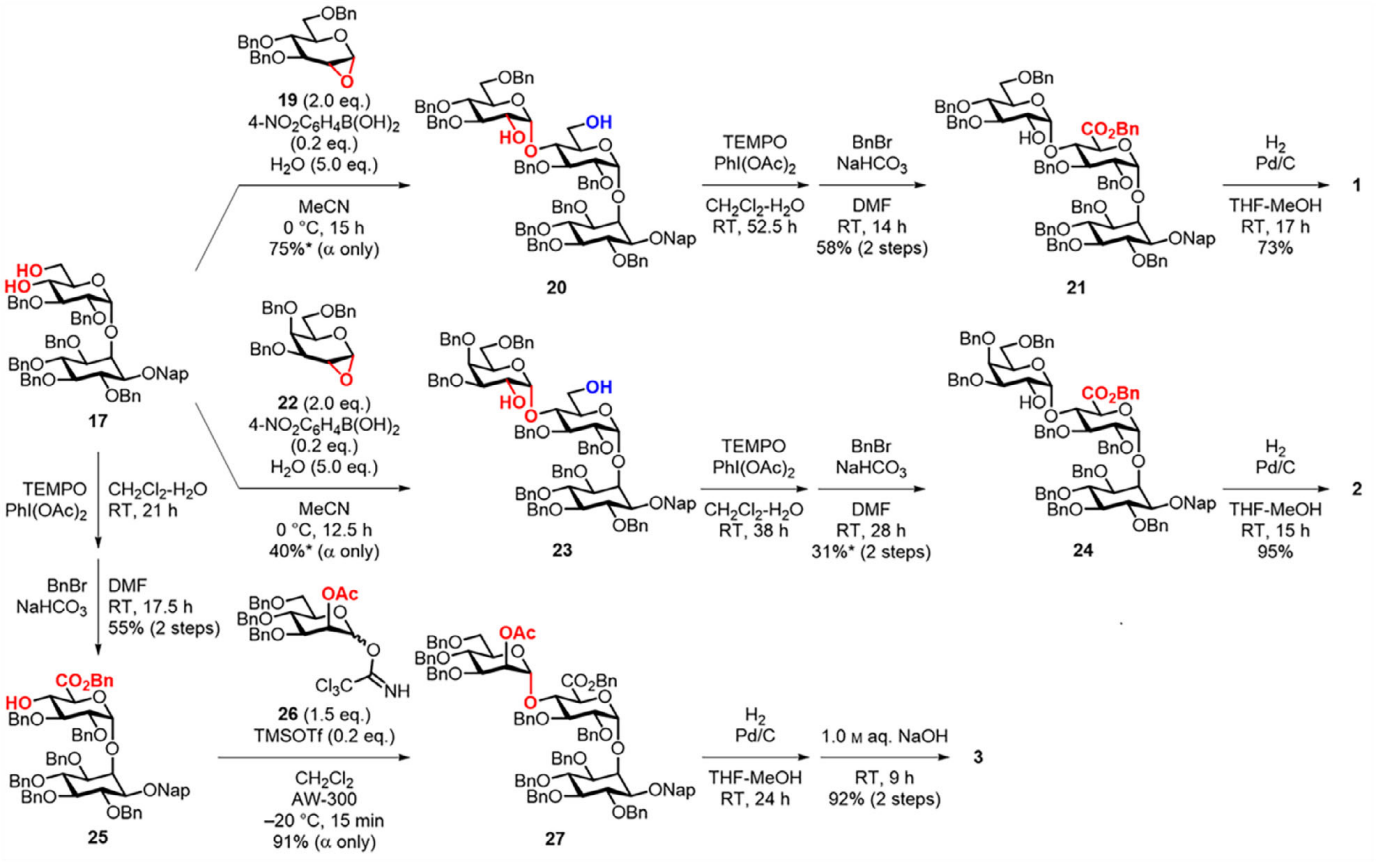
Synthesis of IGs **1**–**3**. *Containing trace impurities.

To synthesize Glc/Gal‐type IGs **1** and **2**, we conducted the challenging 1,2‐*cis*‐α‐glycosylation at the O‐4 position of the Glc residue in **17** by the BMAD method (Scheme 3). The 4,6‐diol acceptor **17** was glycosylated in wet acetonitrile with 1,2‐anhydroglucose donor **19**, prepared from the reaction of glucal **S1** and dimethyldioxirane (see Supporting Information), in the presence of catalytic 4‐nitrophenylboronic acid. The glucosylation at room temperature proceeded α‐selectively to give the desired 4‐*O*‐glucosylated product **20** in 75% yield along with a trace, inseparable impurity that was likely the 6‐*O*‐regioisomer. As reported by the Takahashi and Toshima group, the addition of water suppressed the overreaction by deconstructing a boronic ester intermediate.^[^ ^23^ ^]^ Similarly, galactosylation of **17** at 0 °C using 1,2‐anhydrogalactose **22** successfully afforded the corresponding 4‐*O*‐galactosylated product **23** with α‐selectivity in an acceptable yield of 40%. The decrease in the galactosylation yield may be primarily attributed to the decomposition of **22**, which is more readily hydrolyzed than **19**. TEMPO‐mediated selective primary alcohol oxidation of **20** and **23** followed by benzyl esterification of the resulting carboxylic acids produced protected pseudotrisaccharides **21** and **24**, respectively. Subsequent hydrogenolysis completed the synthesis of **1** and **2**. Toward the synthesis of Man‐type IG **3**, we performed 1,2‐*trans*‐α‐mannosylation of GlcA‐Ins acceptor **25** with 2‐*O*‐acetyl Man donor **26**. After the two‐step conversion of **17** into **25**, the mannosylation using **26** at − 20 °C in the presence of catalytic TMSOTf afforded **27** in a high yield of 92% with complete α‐selectivity. Finally, hydrogenolysis followed by hydrolysis using 1.0 m aq. NaOH yielded **3**.

**Figure 2:**
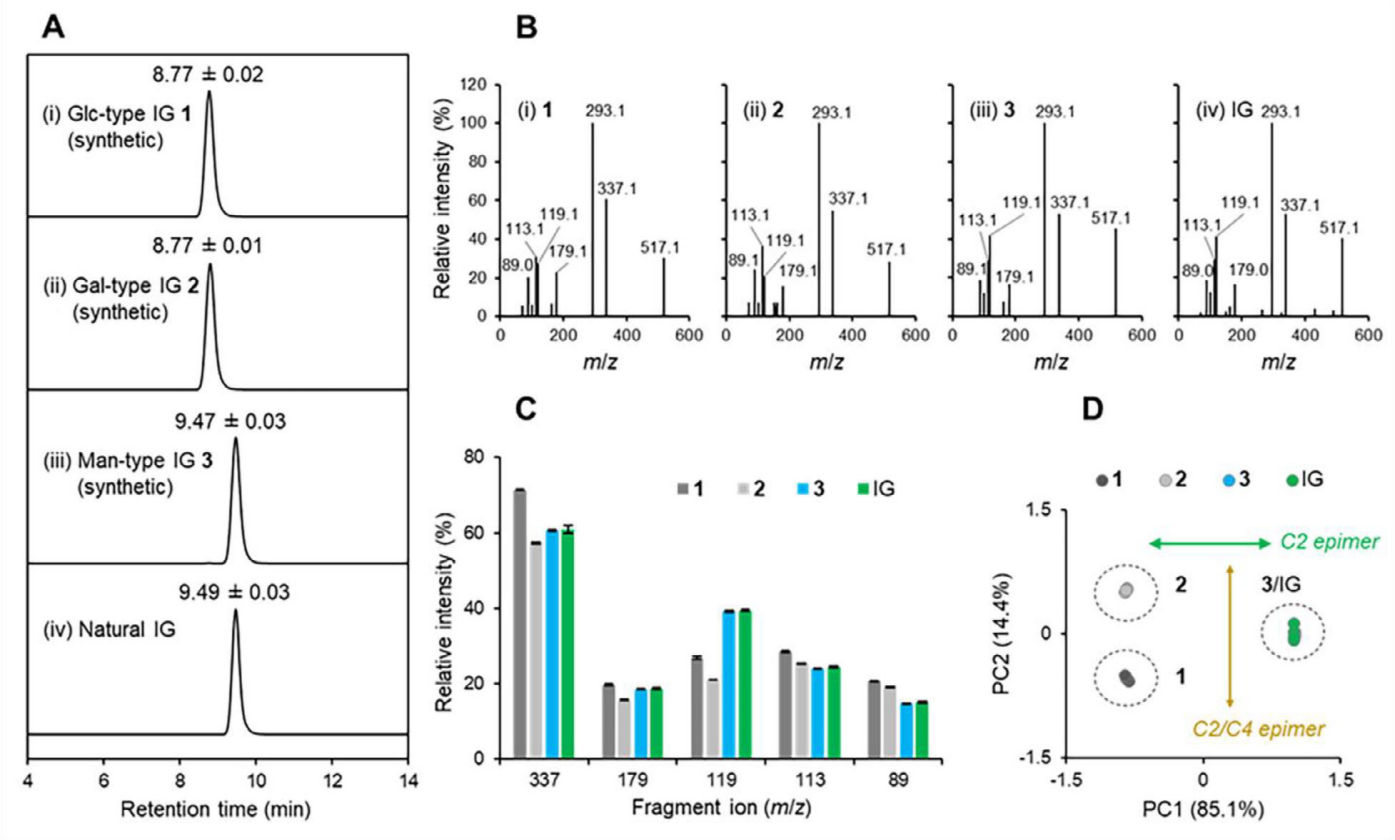
LC‐MS/MS analysis of synthetic IGs **1**–**3** and natural IG. A) LC‐MS/MS chromatograms: (i) **1**, (ii) **2**, (iii) **3**, and (iv) natural IG. [Conditions] Column: ZIC‐pHILIC (2.1 mm I.D. × 150 mm); temperature: 45 °C; flow rate: 0.2 mL/min; MRM transition: *m*/*z* 517.1 > 293.1; eluent: A: 0.02 m NH_4_HCO_3_ (pH 9.8), B: MeCN; gradient: A/B = 25/75 (0–1 minute), 25/75 to 50/50 (1–15 minutes). B) MS/MS spectra: (i) **1**, (ii) **2**, (iii) **3**, and (iv) natural IG. [Conditions] Collision energy: 24 V with Ar gas; N_2_ gas nebulizer: 1.5 L/min; drying: 10 L/minute; temperature: 250 °C; heat block temperature: 300 °C. C) Relative signal intensity of MS^2^ fragments from the precursor ion (*m*/*z* 517.1) obtained by the targeted MRM mode; signal intensity of the primary fragment at *m*/*z* 293.1 was set to 100%. D) Principal component analysis of the MS/MS spectra in Figure 2C.

**Figure 3:**
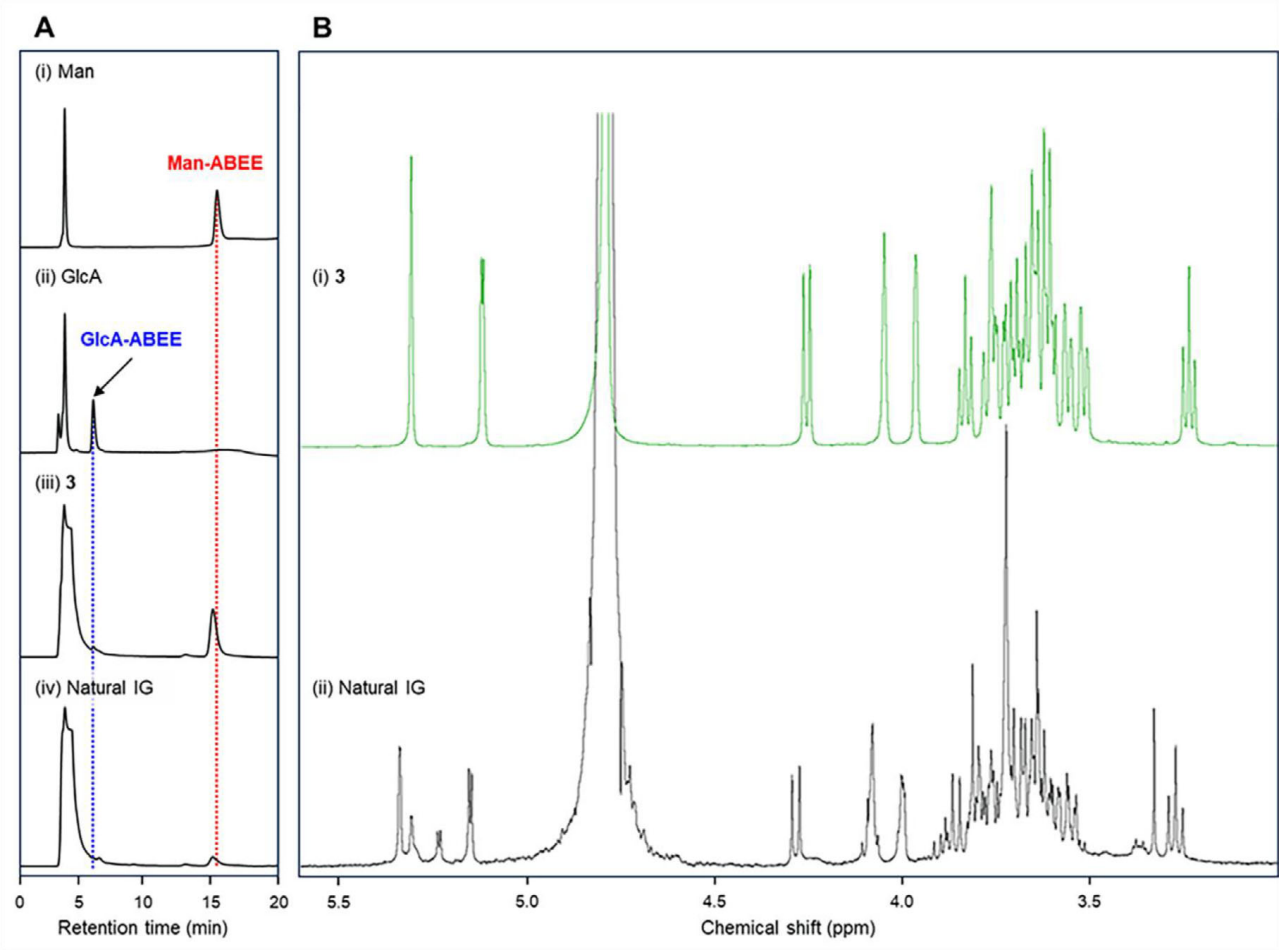
Monosaccharide composition and ^1^H NMR analyses of synthetic IG **3** and natural IG. A) HPLC chromatograms after acid hydrolysis followed by ABEE labeling: (i) Man, (ii) GlcA, (iii) **3**, and (iv) natural IG. [Conditions]: Column: Shodex Asahipak NH2P‐50 4E (4.6 × 250 mm); temperature: 25 °C; flow rate: 0.8 mL/minute; fetection: Fluorescence (Ex. 305 nm, Em. 360 nm); eluent: A: 93% MeCN in 0.3% AcOH (pH adjusted to 7.0 with NH_4_OH), B: 20% MeCN in 0.3% AcOH (pH adjusted to 7.0 with NH_4_OH); gradient: A/B = 97/3 (0–5 min), 97/3 to 75/25 (5–15 min), 75/25 to 68/32 (15–30 min), 68/32 to 97/3 (30–35 min), 97/3 (35–60 min). B) ^1^H NMR spectra (500 MHz) in D_2_O: (i) **3** and (ii) natural IG.

With synthetic IGs **1**–**3** in hand, we determined the structure of a naturally occurring IG, which was obtained via enzymatic degradation of GIPCs isolated from cabbage using GIPC‐PLD.^[^ ^20^ ^]^ Initially, LC‐MS/MS analysis of natural IG was conducted to compare its retention time with that of synthetic standards (Figure 2A). Natural IG was detected with a peak at 9.49 min, which matched with the retention time of Man‐type IG **3** (9.47 min). Peaks for Glc‐ and Gal‐type IGs **1** and **2** were detected at 8.77 min. The corresponding MS/MS spectra are also shown in Figure 2B. As expected, the fragmentation pattern of natural IG closely matched that of **3** (Figure 2C). Principal component analysis of the fragmentation patterns indicated that Glc‐, Gal‐, and Man‐type IGs **1**–**3** produced similar but unique MS/MS spectra based on their epimeric structures at C2 and C4 (Figure 2D). Monosaccharide composition analysis was performed by acid hydrolysis and subsequent labeling with 4‐aminobenzoic acid ethyl ester (ABEE) (Figure 3A).^[^ ^28^ ^]^ Man as well as GlcA was detected as ABEE‐labeled derivatives in both natural IG and **3**. To confirm enzymatically the anomeric stereochemistry and linkage position of the terminal Man moiety in natural IG, we tested Man trimming of **3** using α‐mannosidase in the monosaccharide composition analysis (Figure S 1). Although several α‐mannosidases were evaluated, no Man trimming was observed because they were unable to recognize the structure of d‐Man‐α(1→4)‐d‐GlcA as a suitable substrate. Finally, a comparative analysis of the ^1^H NMR spectra of natural IG and **3** revealed that the signals of the major component of natural IG were in good agreement with that of **3** (Figure 3B). The characteristic signals of the minor component at 5.23 ppm (dd, *J* = 3.8 Hz) and 5.32 ppm (dd, *J* = 1.3 Hz) corresponded to two anomeric protons, indicating it to be either a regio‐ or stereoisomer of **3**. Although the minor component remains unidentified, this evidence strongly supports that the major component of natural IG is d‐Man‐α(1→4)‐d‐GlcA‐α(1→2)‐*myo*‐Ins.

## 3 Conclusion

We successfully synthesized Glc‐type IG **1**, Gal‐type IG **2**, and Man‐type IG **3**. A naturally occurring IG was prepared by isolating GIPCs from cabbage and subjecting them to GIPC‐PLD‐catalyzed degradation. Following this, the structure was determined using LC‐MS/MS, MS/MS spectroscopy, monosaccharide composition, and ^1^H NMR analyses. For the first time, the major component of natural IG was experimentally determined to be d‐Man‐α(1→4)‐d‐GlcA‐α(1→2)‐*myo*‐Ins based on the analytical data obtained. This study paves the way for the chemical synthesis of naturally occurring IGs and GIPCs, thereby facilitating the elucidation of their functions at the molecular level. Efforts to synthesize HexN/HexNAc‐type IGs, structurally diverse GIPCs, and chemical probes for chemical biology studies are currently underway in our laboratory.

## 4 Experimental Section

### General methods

All reactions were carried out under an argon atmosphere unless otherwise noted. All reactions that required heating were performed in an oil bath. All chemicals were purchased from commercial suppliers and used without further purification. Molecular sieves were purchased from FUJIFILM Wako Pure Chemicals (Osaka, Japan) and preactivated at 300 °C for 2 hours in a muffle furnace and then dried in a flask at 300 °C for 2 hours in vacuo prior to use. Dry solvents for reaction media (CH_2_Cl_2_, toluene, THF, CH_3_CN, DMF, and pyridine) were purchased from Kanto Chemical (Tokyo, Japan) and used without purification. TLC analyses were performed using TLC plates (silica gel 60F254 on a glass plate; Merck KGaA, Darmstadt, Germany). Compound detection was carried out either by exposure to UV light (2536 Å) or by soaking in H_2_SO_4_ solution (10% in EtOH) or phosphomolybdic acid solution (20% in EtOH) followed by heating. Flash column chromatography separations were performed by using Biotage Isolera equipped with Biotage Sfär Silica Cartridges. Sephadex LH‐20 (Cytiva, Marlborough, MA, USA) was used for size‐exclusion chromatography. Solvent systems for chromatography were specified in v/v ratios. ^1^H and ^13^C NMR spectra were recorded with AVANCE III 500 and 600 MHz spectrometers (Bruker, Billerica, MA, USA). Chemical shifts in the ^1^H NMR spectra were expressed in ppm (δ) relative to the Me_4_Si signal (0.00 ppm). Chemical shifts in ^13^C NMR spectra were referenced to the residual solvent signal (CDCl_3_, 77.16 ppm) as an internal standard. Data are presented as follows: chemical shift multiplicity (s = singlet, br s = broad singlet, d = doublet, br d = broad doublet, dd = doublet of doublets, t = triplet, m = multiplet), coupling constant (Hz), and integration. Structural assignments were made with additional information from 2D NMR (COSY, HMBC, and HMQC) experiments. High‐resolution mass (ESI‐TOF MS) spectra were run in a Bruker micrOTOF mass spectrometer. Optical rotations were measured with a SEPA‐500 automatic polarimeter (Horiba, Kyoto, Japan). LC‐MS/MS analysis was performed on a LCMS‐8030 system (Shimadzu, Kyoto, Japan). A ZIC‐pHILIC (5.0 µm, 2.1 mm I.D. × 150 mm, Merck) was used as the LC‐MS/MS analytical column. IGs were detected by selected ion monitoring using [M − H]^−^ at *m*/*z* 517.1 followed by product ion scanning or by targeted detection of the product ions. HPLC analysis was performed on an Elite LaChrom HPLC system (Hitachi High‐Tech, Tokyo, Japan) equipped with an L‐2480 Fluorescence Detector (Hitachi High‐Tech, Tokyo, Japan). A Shodex Asahipak NH2P‐50 4E (5.0 µm, 4.6 mm I.D. × 250 mm, Shoko Science) was used as the HPLC analytical column.

## Supporting Information

The authors have cited additional references within the Supporting Information.^[^ ^29^, ^30^, ^31^, ^32^, ^33^, ^34^, ^35^, ^36^ ^]^

## Conflict of Interest

The authors declare no conflict of interest.

## Supporting information



Supporting Information

## Data Availability

The data that support the findings of this study are available in the supplement material of this article.
