# Supplementary material for: Chemical Synthesis and Structural Determination of the Inositol Glycan Head of Plant Sphingolipid GIPC in Brassicaceae
Source: Chemistry. 2025 Jul 25;31(55):e01987. doi: 10.1002/chem.202501987 (PMC12498067; doi:10.1002/chem.202501987)
Supplement: Supplementary file 1 — Supporting Information [file CHEM-31-e01987-s001.pdf]

Table of Contents

1. **Preparation of natural IG sample** ··· S1

2. **LC-MS/MS analysis** ··· S1

3. **Monosaccharide composition analysis** ··· S2–S4

4. **Synthetic procedure** ··· S5–S16

5. **References** ··· S17

6. **NMR spectra of synthetic compounds** ··· S18–S42

1. Preparation of natural IG sample

The natural IG sample was prepared from cabbage GIPC by the GIPC-PLD reaction.[1] The purified GIPC and partially purified GIPC-PLD used in this experiment were freshly prepared from cabbage leaves, as described in our previous reports.[2,3] A typical GIPC-PLD assay mixture consisted of purified GIPC (26 nmol), purified GIPC-PLD fraction (0.04–0.05 mL), and sodium deoxycholate (2.0 mg, 4.8 μmol) in 0.05 M Tris-HCl buffer (pH 7.5) with a total volume of 0.90 mL. The reaction was performed at 30 °C at continuous stirring for 30 min. After heating at 80 °C for 5 min for enzyme inactivation, the reaction mixture was subjected to two-phase separation consisted with $CHCl_3$/MeOH/H_2O (1:1:0.9, v/v/v). The upper layer was taken, dried and washed with EtOH. The residue was separated by preparative TLC developed with 5% aq. NH_3/EtOH (25:50, v/v). The band corresponding to the IG was scraped from the TLC plate, and the collected silica gel was extracted with H_2O. The resulting extract was concentrated in vacuo, and the residue was washed with 25% aq. MeOH. The remaining material was used as the natural IG sample.

2. LC-MS/MS analysis

Liquid chromatography-electrospray ionization-tandem mass spectrometry (LC-ESI-MS/MS) analysis was performed using LCMS-8030 system (Shimadzu, Kyoto, Japan) equipped with ZIC-pHILIC column (5.0 μm, 2.1 mm I.D. × 150 mm, Merck) kept at 45 ºC. Solvent A (0.02 M NH_4HCO_3, pH 9.8) and solvent B (MeCN) were used in the following gradient conditions at a flow rate of 0.2 mL/min: 0 min, 75% B and kept for 1 min; 1–15 min, 75–50% B and kept for 5 min; 15–20 min, 50–75% B and kept for 10 min for pre-conditioning. Synthetic IGs **1**–**3** and natural IG were detected by selected ion monitoring using $[M-H]^-$ at m/z 517.1 and the product ions were scanned between *m/z* 50 and 520 at 24 V of collision energy. The other MS conditions were as follows: capillary voltage, 4.5 kV; desolvation gas (N_2) flow, 10 L/min; nebulizer gas (N_2) flow, 1.5 L/min; conversion dynode voltage, 6 kV; source temperature, 250◦C; and collision gas (Ar) flow, 230 kPa.

3. Monosaccharide composition analysis

I. Method using acid hydrolysis followed by ABEE labeling

To determine the monosaccharide composition of the natural IG and the synthetic Man-type IG standard **3**, each sample (10 µL, 0.5 × 10^{-3} M in H_2O) was mixed with an equal volume of 2 M hydrochloric acid (final concentration: 1 M HCl) and heated at 100 °C for 3 h in sealed tubes. After hydrolysis, the samples were dried under reduced pressure and reconstituted in 10 µL of Milli-Q H_2O. For derivatization, 40 µL of freshly prepared ABEE labeling reagent (165 mg of ethyl 4-aminobenzoate and 35 mg of sodium cyanoborohydride dissolved in 41 µL of AcOH and 350 µL of MeOH at 60 °C) was added to each sample and incubated at 80 °C for 60 min. After cooling, 100 µL of Milli-Q H_2O and 200 µL of $CHCl_3$ were added, followed by vortexing and centrifugation at 14,000 × *g* for 5 min.[4] The upper aqueous phase, which contained ABEE-labeled monosaccharides, was collected and subsequently analyzed by HPLC (Figure 3A).

II. Method using α-mannosidase treatment followed by ABEE labeling

A) Plasmid construction and expression of α-mannosidase Bt4073

A codon-optimized cDNA encoding residues 29–782 of the α-mannosidase Bt4073 from *Bacteroides thetaiotaomicron* (GenBank accession number: AAO79178) was chemically synthesized by Eurofins Genomics (Tokyo, Japan) for heterologous expression in *Escherichia coli*. The gene was inserted into the SmaI site of the pET15-SmaI vector using the In-Fusion HD Cloning Kit (Takara Bio, Shiga, Japan), resulting in the recombinant plasmid pET15-SmaI-Bt4073, which encodes Bt4073 with an N-terminal 6×His tag.[5] The construct was transformed into *E. coli* SHuffle T7 Express (New England Biolabs, MA, USA). Protein expression was induced according to the standard protocol provided with the SHuffle T7 Express system, and the recombinant protein was purified using His60 Ni Superflow Resin (Takara Bio) following the manufacturer’s instructions.

B) Substrate specificity of Bt4073

Four synthetic mannobiose isomers with α-(1→2)-, α-(1→3)-, α-(1→4)-, and α-(1→6)-linkages (Dextra Laboratories Ltd., Reading, United Kingdom) were used to evaluate the substrate specificity of Bt4073. Each substrate (1×10^{-3} M final concentration) was incubated with purified Bt4073 (1.0 µg) in 0.1 M MOPS buffer (pH 7.0) containing 2×10^{-3} M $CaCl_2$ at 37 °C for 4 h (total volume: 20 µL). Reactions were terminated by heating at 95 °C for 5 min and cooling on ice. 10 µL of each reaction mixture were subjected to ABEE labeling as described above. Labeled saccharides were analyzed by HPLC (Figure S1A–D).

C) α-Mannosidase treatment followed by ABEE labeling of the synthetic Man-type IG **3**

To examine whether the synthetic Man-type IG **3** is susceptible to enzymatic cleavage by Bt4073, each sample (10 µL, 0.50×10^{-3} M in H_2O) was incubated with Bt4073 (1.0 µg) under the same reaction conditions described above (0.1 M MOPS buffer, pH 7.0, 2×10^{-3} M $CaCl_2$, 37 °C, 4 h). Reactions were terminated by heating at 95 °C for 5 min and cooling on ice. 10 µL of each digested mixture were subjected to ABEE labeling, and released monosaccharides were analyzed by HPLC (Figure S1E).

D) HPLC conditions

All ABEE-labeled samples were analyzed using an Elite LaChrom HPLC system (Hitachi High-Tech, Tokyo, Japan) equipped with an L-2480 fluorescence detector. A Shodex Asahipak NH2P-50 4E amino column (5.0 µm, 4.6 mm I.D. × 250 mm, Showa Denko, Tokyo, Japan) was used at 25 °C. The mobile phases consisted of solvent A (93% acetonitrile in 0.3% NH_4OAc, pH adjusted to 7.0 with aq. NH_3) and solvent B (20% acetonitrile in 0.3% NH_4OAc, pH adjusted to 7.0 with aq. NH_3). The gradient program was as follows (flow rate: 0.8 mL/min): 0–5 min, isocratic 3% B; 5–15 min, linear increase to 25% B; 15–30 min, to 32% B; 30–35 min, back to 3% B; and 35–60 min, isocratic 3% B. Detection of ABEE-labeled sugars was performed using fluorescence with excitation at 305 nm and emission at 360 nm.

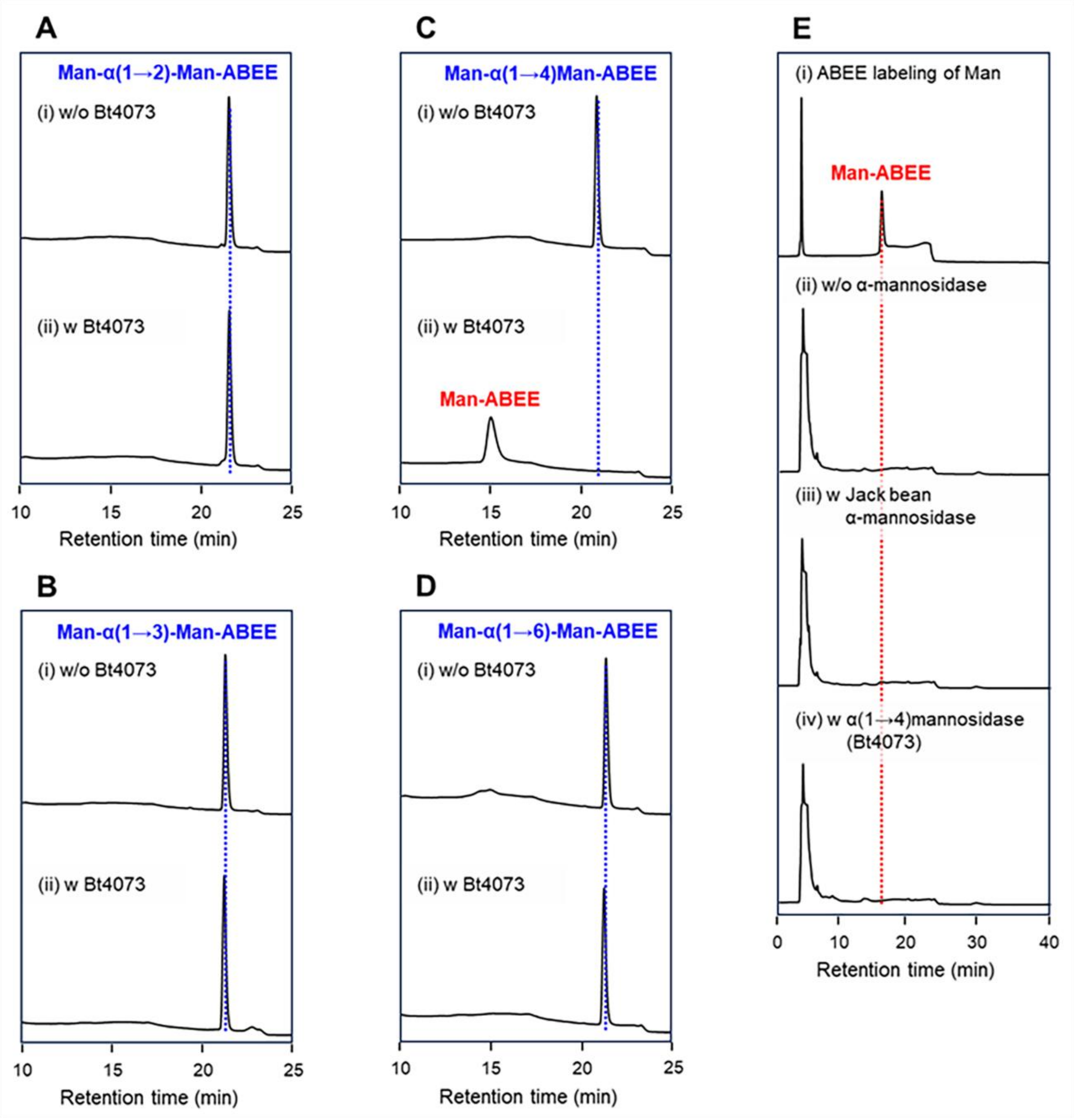

Figure S1. Substrate specificity and IG digestion activity of Bt4073. (A–D) HPLC analysis of ABEE-labeled products following incubation of Bt4073 with four synthetic mannobiose isomers: (A) α-(1→2)-mannobiose, (B) α-(1→3)-mannobiose, (C) α-(1→4)-mannobiose, and (D) α-(1→6)-mannobiose. Each substrate (1×10^{-3} M) was reacted with purified Bt4073 (1.0 µg) in 0.1 M MOPS buffer (pH 7.0) containing 2×10^{-3} M $CaCl_2$ at 37 °C for 4 h, followed by ABEE labeling and fluorescence detection. Only the α-(1→4)-linked mannobiose was cleaved, indicating that Bt4073 exhibits strict specificity for the α-(1→4) linkage. (E) HPLC analysis of ABEE-labeled monosaccharides released from the synthetic Man-type IG **3** after enzymatic treatment. (i) ABEE-labeled mannose (standard control); (ii) untreated sample (no enzyme control); (iii) sample treated with Jack Bean α-mannosidase; (iv) sample treated with Bt4073.

4. Synthetic procedure

I. General methods

All reactions were carried out under an argon atmosphere unless otherwise noted. All reactions that required heating were performed in an oil bath. All chemicals were purchased from commercial suppliers and used without further purification. 3,4,6-Tri-*O*-benzyl-D-glucal **S1** were purchased from Tokyo Chemical Industry (Tokyo, Japan). 3,4,6-Tri-*O*-benzyl-D-glactal **S2**,[6] 1D-3,4,5,6-tetra-*O*-benzyl-*myo*-inositol **14**,[7] ethyl 2,3-di-*O*-benzyl-4,6-*O*-phenylboronate-1-thio-β-D-glucopyranoside **16**,[8] 1,2-anhydro-3,4,6-tri-*O*-benzyl-D-glucopyranose **19**,[9] 1,2-anhydro-3,4,6-tri-*O*-benzyl-D-galactopyranose **22**,[10] 2-*O*-acetyl-3,4,6-tri-*O*-benzyl-D-mannopyranosyl trichloroacetimidate **26**[11] were prepared using previously reported procedures with minor modifications. Molecular sieves were purchased from FUJIFILM Wako Pure Chemicals (Osaka, Japan) and pre-dried at 300 °C for 2 h in a muffle furnace and then dried in a flask at 300 °C for 2 h in vacuo prior to use. Dry solvents for reaction media (CH_2Cl_2, toluene, THF, CH_3CN, DMF, MeOH, and pyridine) were purchased from Kanto Chemical (Tokyo, Japan) and used without purification. TLC analyses were performed using TLC plates (silica gel 60F254 on a glass plate; Merck KGaA, Darmstadt, Germany). Compound detection was carried out either by exposure to UV light (253.6 nm) or by soaking in H_2SO_4 solution (10% in EtOH) or solution phosphomolybdic acid solution (20% in EtOH) followed by heating. Flash column chromatography separations were performed by using Biotage Isolera equipped with Biotage Sfär Silica Cartridges. Sephadex LH-20 (Cytiva, Marlborough, MA, USA) was used for size-exclusion chromatography. Solvent systems for chromatography were specified in v/v ratios. ^1H and ^{13}C NMR spectra were recorded with a AVANCE III 500 and 600 spectrometers (Bruker, Billerica, MA, USA). Chemical shifts in ^1H NMR spectra were expressed in ppm (δ) relative to the Me_4Si signal (0.00 ppm). Chemical shifts in ^{13}C NMR spectra were reported to the residual solvent signal ($CDCl_3$, 77.16 ppm) as an internal standard. Data are presented as follow: chemical shift multiplicity (s = singlet, br s = broad singlet, d = doublet, br d = broad doublet, dd = doublet of doublets, t = triplet, m = multiplet), coupling constant (Hz), and integration. Each of the Glc units in **20** is labeled with letters: a for the reducing end and b for non-reducing end. Structural assignments were made with additional information from 2D NMR (COSY, HMBC, and HMQC) experiments. High-resolution mass (ESI-TOF MS) spectra were run in a Bruker micrOTOF mass spectrometer. Optical rotations were measured with a SEPA-500 automatic polarimeter (Horiba, Kyoto, Japan).

II. Synthesis of glucosyl inositol **17**

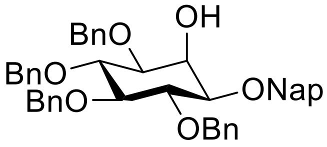

1D-3,4,5,6-Tetra-*O*-benzyl-1-*O*-(2-naphthalenylmethyl)-*myo*-inositol (15)

To a solution of 1D-3,4,5,6-tetra-*O*-benzyl-*myo*-inositol **14**[7] (2.10 g, 3.88 mmol) in toluene (97.1 mL) was added dibutyltin oxide (967 mg, 3.88 mmol) at room temperature. After 3 h of stirring under reflux, the reaction mixture was concentrated in vacuo. The resulting residue was dissolved in toluene (38.8 mL). To the solution were added 2-(bromomethyl)naphthalene (1.72 g, 7.76 mmol) and tetrabutylammonium bromide (625 mg, 1.94 mmol) at room temperature and the reaction mixture was stirred at 70 °C. The progress of the reaction was monitored by TLC (*n*-hexane/EtOAc = 2:1). After 15 h of stirring, the reaction mixture was concentrated in vacuo. The resulting residue was purified using silica gel column chromatography (toluene/EtOAc = 95:5) to yield **15** (2.01 g, 76%, 2 steps) as a white solid. $[\alpha]_D^{20}$ +1.9° (c 1.1, $CHCl_3$); ^1H NMR (500 MHz, $CDCl_3$) δ 7.85–7.23 (m, 27 H, Ar), 4.96–4.65 (m, 10 H, 10 ArCH_2), 4.22 (t, 1 H, $J_{1,2}$ = $J_{2,3}$ = 2.6 Hz, H-2), 4.04 (t, 1 H, $J_{1,6}$ = $J_{5,6}$ = 9.5 Hz, H-6), 4.00 (t, 1 H, $J_{3,4}$ = $J_{4,5}$ = 9.5 Hz, H-4), 3.49–3.41 (m, 2 H, H-1, H-5), 3.37 (dd, 1 H, H-3), 2.51 (s, 1 H, 2-OH); ^{13}C NMR (125 MHz, $CDCl_3$) δ 138.8, 138.7, 138.7, 137.9, 135.4, 133.2, 133.0, 128.4, 128.4, 128.3, 128.3, 128.0, 127.9, 127.9, 127.8, 127.7, 127.6, 126.7, 126.2, 126.0, 125.9, 83.2, 81.2, 79.7, 79.6, 76.0, 75.9, 72.8, 72.7, 67.6; HRMS (ESI) *m/z*: found $[M+Na]^+$ 703.3032, $C_{45}H_{44}O_6$ calcd for $[M+Na]^+$ 703.3030.

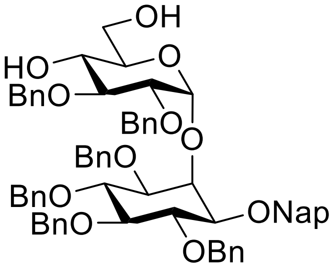

2,3-Di-*O*-benzyl-α-D-glucopyranosyl-(1→2)-1D-3,4,5,6-tetra-*O*-benzyl-1-*O*-(2-naphthalenylmethyl)-*myo*-inositol (17)

15 (380 mg, 558 μmol) and ethyl 2,3-di-*O*-benzyl-4,6-*O*-phenylboronate-1-thio-β-D-glucopyranoside **16**[8] (250 mg, 507 μmol) were mixed in a pear-shaped flask, and then residual H_2O was azeotropically removed with dry toluene. After exposure to high vacuum overnight, the mixture was dissolved in CH_2Cl_2 (20.3 mL) and the solution was then

transferred to a two-necked flask containing pre-activated molecular sieves 3Å (2.03 g) via cannula. After 15 min of stirring at room temperature, MeOTf (139 µL, 1.27 mmol) was added to the mixture and then heated to reflux. The progress of the reaction was monitored by TLC (toluene/acetone = 4:1). After 4 h of stirring under reflux, the reaction mixture was filtered through a pad of Celite®, which was rinsed with EtOAc. The combined filtrate was washed with 1.0 M aq. solution of sorbitol and Na_2CO_3 and brine. The organic layer was dried over Na_2SO_4, filtered off, and concentrated in vacuo. The resulting residue was purified using size-exclusion chromatography on Sephadex LH-20 ($CHCl_3$/MeOH = 1:1) and flash silica gel column chromatography (toluene/EtOAc = 90:10 → 85:15) to yield **17** (425 mg, 82%) as a white powder.

$[\alpha]_D^{20}$ +40.0° (c 0.9, $CHCl_3$); ^{1}H NMR (500 MHz, $CDCl_3$) δ 7.87–6.94 (m, 37 H, Ar), 5.61 (d, 1 H, $J_{1,2}$ = 3.5 Hz, H-1^{Glc}), 5.09–4.56 (m, 13 H, 13 ArCH_2), 4.45–4.39 (m, 2 H, ArCH_2, H-2^{Ins}), 4.22–4.14 (m, 2 H, H-6^{Ins}, H-5^{Glc}), 4.04 (t, 1 H, $J_{3,4}$ = $J_{4,5}$ = 9.6 Hz, H-4^{Ins}), 3.82 (t, 1 H, $J_{2,3}$ = $J_{3,4}$ = 9.3 Hz, H-3^{Glc}), 3.56–3.40 (m, 6 H, H-2^{Glc}, H-4^{Glc}, H-6a^{Glc}, H-6b^{Glc}, H-1^{Ins}, H-5^{Ins}), 3.38 (dd, 1 H, $J_{2,3}$ = 2.3 Hz, H-3^{Ins}), 2.29 (d, 1 H, $J_{4,OH}$ = 2.5 Hz, 4-OH^{Glc}), 1.47 (dd, 1 H, J = 5.0 Hz, J = 7.7 Hz, 6-OH^{Glc}); ^{13}C NMR (125 MHz, $CDCl_3$) δ 139.1, 138.9, 138.8, 138.6, 138.2, 138.0, 135.2, 133.3, 133.1, 128.5, 128.4, 128.4, 128.4, 128.3, 128.2, 128.1, 128.0, 128.0, 127.7, 127.7, 127.7, 127.5, 127.4, 127.4, 127.3, 127.2, 127.1, 126.9, 126.3, 126.3, 126.2, 96.7, 83.9, 81.6, 81.5, 81.4, 80.1, 79.4, 79.1, 76.2, 75.8, 75.4, 75.0, 74.0, 72.7, 72.0, 71.1, 70.4, 70.3, 62.4; HRMS (ESI) *m/z*: found $[M+Na]^+$ 1045.4502, $C_{65}H_{66}O_{11}$ calcd for $[M+Na]^+$ 1045.4497.

III. Synthesis of Glc-type inositol glycan **1**

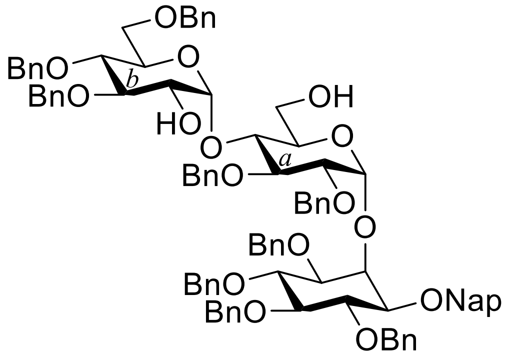

3,4,6-Tri-*O*-benzyl-α-D-glucopyranosyl-(1→4)-2,3-di-*O*-benzyl-α-D-glucopyranosyl-(1→2)-1D-3,4,5,6-tetra-*O*-benzyl-1-*O*-(2-naphthalenylmethyl)-*myo*-inositol (20)

To a solution of 3,4,6-tri-*O*-benzyl-D-glucal **S1** (31.0 mg, 73.3 µmol) in CH_2Cl_2 (730 µL) was added dimethyldioxirane[12] (0.06 M solution in acetone: 1.5 mL, 88 µmol) at 0 °C and the

reaction mixture was stirred at the same temperature. The progress of the reaction was monitored by TLC (*n*-hexane/EtOAc = 4:1). After 15 min of stirring, the reaction mixture was concentrated in vacuo and exposed to high vacuum. The resulting 1,2-anhydroglucose **19** was dissolved in in CH_3CN (600 µL). The solution was added to a solution of **17** (25.0 mg, 24.4 µmol), 4-nitrophenylboronic acid (0.8 mg, 4.9 µmol), H_2O (2.2 µL, 122 µmol) in CH_3CN (130 µL) at 0 °C and the reaction mixture was stirred at the same temperature. The progress of the reaction was monitored by TLC (*n*-hexane/acetone = 2:1). After 15 h of stirring, the reaction was quenched by addition of 0.05 M aq. $NaBO_3$ (2.0 mL, 98.0 µmol) and extracted with EtOAc. The organic layer was washed with sat. aq. NH_4Cl and brine, dried over Na_2SO_4, filtered off, and concentrated in vacuo. The resulting residue was purified using size-exclusion chromatography on Sephadex LH-20 ($CHCl_3$/MeOH = 1:1) and flash silica gel column chromatography (*n*-hexane/acetone = 70:30) to yield **20** (27.0 mg, 75%, α only) containing trace impurities as a white powder.

^{1}H NMR (500 MHz, $CDCl_3$) δ 7.88–6.81 (m, 52 H, Ar), 5.64 (d, 1 H, $J_{1,2}$ = 3.5 Hz, H-1$^{Glc\text{-}a}$), 5.23 (d, 1 H, $J_{1,2}$ = 3.9 Hz, H-1$^{Glc\text{-}b}$), 5.21–4.34 (m, 21 H, H-2^{Ins}, 20 ArC*H*$_2$), 4.18 (t, 1 H, $J_{1,6}$ = $J_{5,6}$ = 9.5 Hz, H-6^{Ins}), 4.14–4.04 (m, 2 H, H-5$^{Glc\text{-}a}$, H-4^{Ins}), 3.97 (t, 1 H, $J_{2,3}$ = $J_{3,4}$ = 9.4 Hz, H-3$^{Glc\text{-}a}$), 3.80 (t, 1 H, $J_{4,5}$ = 9.6 Hz, H-4$^{Glc\text{-}a}$), 3.77–3.34 (m, 12 H, H-2$^{Glc\text{-}a}$, H-6a$^{Glc\text{-}a}$, H-6b$^{Glc\text{-}a}$, H-2$^{Glc\text{-}b}$, H-3$^{Glc\text{-}b}$, H-4$^{Glc\text{-}b}$, H-5$^{Glc\text{-}b}$, H-6a$^{Glc\text{-}b}$, H-6b$^{Glc\text{-}b}$, H-1^{Ins}, H-3^{Ins}, H-5^{Ins}), 3.08 (d, 1 H, $J_{2,OH}$ = 8.9 Hz, 2-OH$^{Glc\text{-}b}$), 2.54 (br s, 1 H, 6-OH$^{Glc\text{-}a}$); ^{13}C NMR (125 MHz, $CDCl_3$) δ 139.0, 138.8, 138.8, 138.6, 138.2, 138.1, 138.0, 137.8, 137.5, 135.1, 133.3, 133.2, 128.4, 128.4, 128.4, 128.4, 128.3, 128.3, 128.3, 128.2, 128.1, 128.0, 128.0, 127.9, 127.9, 127.9, 127.8, 127.7, 127.7, 127.7, 127.6, 127.5, 127.5, 127.4, 127.4, 127.3, 127.2, 127.0, 126.3, 126.3, 126.2, 101.3 (C-1$^{Glc\text{-}b}$), 96.3 (C-1$^{Glc\text{-}a}$), 83.9, 83.3, 81.5, 81.5, 81.2, 79.8, 79.7, 79.0, 77.2, 76.2, 75.6, 75.4, 75.1, 75.0, 74.7, 74.0, 73.5, 73.4, 72.6, 71.7, 71.6, 70.9, 70.6, 68.8, 60.7; HRMS (ESI) *m/z*: found [M+Na]$^+$ 1477.6445, $C_{92}H_{94}O_{16}$ calcd for [M+Na]$^+$ 1477.6434.

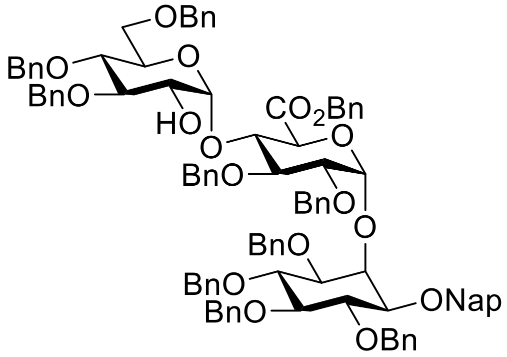

3,4,6-Tri-O-benzyl-α-D-glucopyranosyl-(1→4)-benzyl 2,3-di-*O*-benzyl-α-D-glucopyranosyluronate-(1→2)-1D-3,4,5,6-tetra-*O*-benzyl-1-*O*-(2-naphthalenylmethyl)-

***myo*-inositol (21)**

To a solution of **20** (20.0 mg, 13.7 μmol) in CH_2Cl_2 (1.3 mL) were added TEMPO (2.4 mg, 15.1 μmol), H_2O (0.5 mL), and iodobenzene diacetate (8.9 mg, 27.5 μmol) at room temperature and the reaction mixture was stirred at the same temperature. The progress of the reaction was monitored by TLC (*n*-hexane/acetone = 2:1). After 4 h of stirring, iodobenzene diacetate (4.5 mg, 13.7 μmol) was added. After 19 h of stirring, iodobenzene diacetate (8.9 mg, 27.5 μmol) was added. An additional 4 h of stirring was followed by the addition of iodobenzene diacetate (8.9 mg, 27.5 μmol). After 1 h of stirring, TEMPO (2.4 mg, 15.1 μmol) was added. After 17 h of further stirring, iodobenzene diacetate (8.9 mg, 27.5 μmol) was added. The reaction mixture was stirred for an additional 7.5 h and then concentrated in vacuo. The resulting residue was exposed to high vacuum for 2 h and dissolved in DMF (274 μL). To the solution were added benzyl bromide (6.5 μL, 55 μmol) and $NaHCO_3$ (9.2 mg, 110 μmol) at room temperature and the reaction mixture was stirred at the same temperature. The progress of the reaction was monitored by TLC (*n*-hexane/acetone = 2:1). After 1 h of stirring, $NaHCO_3$ (10.2 mg, 124 μmol) was added. The reaction mixture was stirred for an additional 13 h and diluted with *n*-hexane/EtOAc (4:1) and washed with sat. aq. $NaHCO_3$. The organic layer was dried over Na_2SO_4, filtered off, and concentrated in vacuo. The resulting residue was purified using flash silica gel column chromatography (*n*-hexane/acetone = 87:13) to yield **21** (12.4 mg, 58%, 2 steps) as a colorless syrup.

$[\alpha]_D^{20}$ +41.8° (c 2.9, $CHCl_3$); ^1H NMR (500 MHz, $CDCl_3$) δ 7.87–6.83 (m, 57 H, Ar), 5.67 (d, 1 H, $J_{1,2}$ = 3.5 Hz, H-1^{GlcA}), 5.31 (d, 1 H, $J_{1,2}$ = 2.4 Hz, H-1^{Glc}), 5.15–4.36 (m, 23 H, H-5^{GlcA}, 22 ArC*H*$_2$), 4.34 (br s, 1 H, H-2^{Ins}), 4.11 (t, 1 H, $J_{1,6}$ = $J_{5,6}$ = 9.6 Hz, H-6^{Ins}), 4.04–3.96 (m, 3 H, H-3^{GlcA}, H-4^{GlcA}, H-4^{Ins}), 3.77–3.52 (m, 7 H, H-2^{Glc}, H-3^{Glc}, H-4^{Glc}, H-5^{Glc}, H-6a^{Glc}, H-6b^{Glc}, H-2^{GlcA}), 3.42 (t, 1 H, $J_{4,5}$ = 9.3 Hz, H-5^{Ins}), 3.34 (dd, 1 H, $J_{1,2}$ = 1.4 Hz, H-1^{Ins}), 3.27 (dd, 1 H, $J_{2,3}$ = 2.2 Hz, $J_{3,4}$ = 9.9 Hz, H-3^{Ins}), 2.82 (d, 1 H, $J_{2,OH}$ = 8.7 Hz, 2-OH^{Glc}); ^{13}C NMR (125 MHz, $CDCl_3$) δ 169.8, 139.0, 138.9, 138.8, 138.5, 138.4, 138.0, 138.0, 137.8, 137.6, 135.0, 134.7, 133.2, 133.1, 128.6, 128.4, 128.4, 128.4, 128.3, 128.3, 128.3, 128.2, 128.1, 128.0, 128.0, 127.9, 127.9, 127.8, 127.7, 127.7, 127.6, 127.5, 127.5, 127.4, 127.4, 127.3, 127.2, 127.0, 126.3, 126.2, 100.5, 97.0, 83.8, 83.3, 81.3, 81.2, 80.9, 79.2, 79.1, 76.2, 75.5, 75.3, 75.3, 75.2, 75.0, 74.0, 73.5, 73.3, 72.9, 71.8, 71.3, 71.2, 71.0, 67.9, 67.6; HRMS (ESI) *m/z*: found $[M+Na]^+$ 1581.6701, $C_{99}H_{98}O_{17}$ calcd for $[M+Na]^+$ 1581.6696.

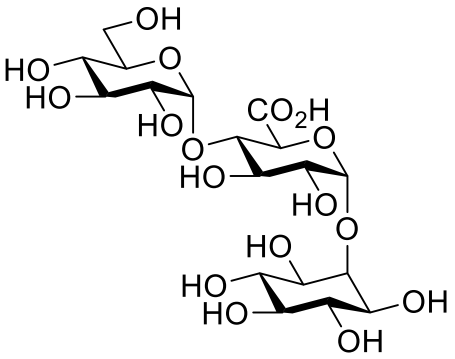

α-D-Glucopyranosyl-(1→4)-α-D-glucopyranosyluronic acid-(1→2)-*myo*-inositol (1)

To a solution of **21** (11.1 mg, 7.12 μmol) in MeOH/THF (3:1, 1.8 mL), Pd/C (10% on carbon: 15.1 mg, 14.2 μmol) was added at room temperature. After degassing and filling with H_2 gas three times, the reaction mixture was stirred at room temperature under H_2 gas atmosphere. The progress of the reaction was monitored by TLC ($CHCl_3$/MeOH/AcOH = 1:2:0.1). After 17 h of stirring, the reaction mixture was filtered through a pad of Celite® and the pad was rinsed with MeOH. The combined filtrate was concentrated in vacuo. The resulting residue was purified using size-exclusion chromatography on Sephadex LH-20 (MeOH/H_2O = 1:1) and lyophilized to yield **1** (2.7 mg, 73%) as a white powder.

^{1}H NMR (500 MHz, D_2O) δ 5.50 (d, 1 H, $J_{1,2}$ = 3.9 Hz, H-1^{Glc}), 5.21 (d, 1 H, $J_{1,2}$ = 3.8 Hz, H-1^{GlcA}), 4.38 (d, 1 H, $J_{4,5}$ = 10.0 Hz, H-5^{GlcA}), 4.14 (t, 1 H, $J_{1,2}$ = $J_{2,3}$ = 2.6 Hz, H-2^{Ins}), 4.01 (t, 1 H, $J_{2,3}$ = $J_{3,4}$ = 9.4 Hz, H-3^{GlcA}), 3.85–3.67 (m, 8 H, H-3^{Glc}, H-5^{Glc}, H-6a^{Glc}, H-6b^{Glc}, H-2^{GlcA}, H-4^{GlcA}, H-4^{Ins}, H-6^{Ins}), 3.66–3.57 (m, 2 H, H-1^{Ins}, H-3^{Ins}), 3.54 (dd, 1 H, $J_{2,3}$ = 9.9 Hz, H-2^{Glc}), 3.42 (t, 1 H, $J_{3,4}$ = $J_{4,5}$ = 9.7 Hz, H-4^{Glc}), 3.31 (t, 1 H, $J_{4,5}$ = $J_{5,6}$ = 9.3 Hz, H-5^{Ins}); ^{13}C NMR (125 MHz, D_2O) δ 176.0, 100.5, 98.4, 81.5, 76.8, 74.5, 73.7, 73.1, 72.9, 72.8, 72.7, 72.0, 71.9, 71.8, 70.4, 69.4, 60.2; HRMS (ESI) *m/z*: found [M−H]$^-$ 517.1411, $C_{18}H_{29}O_{17}$ calcd for [M−H]$^-$ 517.1410.

IV. Synthesis of Gal-type inositol glycan **2**

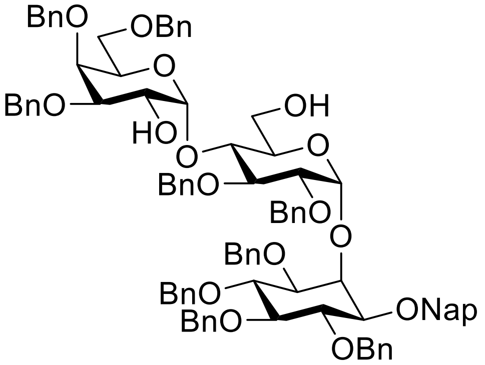

3,4,6-Tri-*O*-benzyl-α-D-galactopyranosyl-(1→4)-2,3-di-*O*-benzyl-α-D-glucopyranosyl-(1→2)-1D-3,4,5,6-tetra-*O*-benzyl-1-*O*-(2-naphthalenylmethyl)-*myo*-inositol (23)

To a solution of 3,4,6-tri-*O*-benzyl-D-galactal **S2**[6] (40.7 mg, 97.7 μmol) in CH_2Cl_2 (977 μL) was added dimethyldioxirane[12] (0.06 M solution in acetone: 1.96 mL, 117 μmol) at 0 °C and the reaction mixture was stirred at the same temperature. The progress of the reaction was monitored by TLC (*n*-hexane/EtOAc = 4:1). After 15 min of stirring, the reaction mixture was concentrated in vacuo and exposed to high vacuum for 1 h. The resulting 1,2-anhydrogalactose **22** was dissolved in in CH_3CN (600 μL) and then the solution was added to a solution of **17** (50.0 mg, 48.9 μmol), 4-nitrophenylboronic acid (1.6 mg, 9.8 μmol), H_2O (4.4 μL, 244 μmol) in CH_3CN (900 μL) at 0 °C and the reaction mixture was stirred at the same temperature. The progress of the reaction was monitored by TLC (*n*-hexane/acetone = 2:1). After 12.5 h of stirring, the reaction was quenched by addition of 0.5 M aq. $NaBO_3$ (0.39 mL, 195 μmol) and extracted with EtOAc. The organic layer was washed with sat. aq. NH_4Cl and brine, dried over Na_2SO_4, filtered off, and concentrated in vacuo. The resulting residue was purified using size-exclusion chromatography on Sephadex LH-20 ($CHCl_3$/MeOH = 1:1) and flash silica gel column chromatography (*n*-hexane/acetone = 80:20) to yield **23** (34.4 mg, 40%, α only) containing trace impurities as a colorless syrup.

^{1}H NMR (500 MHz, $CDCl_3$) δ 7.86–6.86 (m, 52 H, Ar), 5.64 (d, 1 H, $J_{1,2}$ = 3.6 Hz, H-1^{Glc}), 5.45 (d, 1 H, $J_{1,2}$ = 4.3 Hz, H-1^{Gal}), 5.13–4.35 (m, 21 H, H-2^{Ins}, 20 ArCH_2), 4.18 (t, 1 H, $J_{1,6}$ = $J_{5,6}$ = 9.5 Hz, H-6^{Ins}), 4.15–4.05 (m, 3 H, H-2^{Gal}, H-5^{Glc}, H-4^{Ins}), 3.98 (t, 1 H, $J_{2,3}$ = $J_{3,4}$ = 9.4 Hz, H-3^{Glc}), 3.88–3.80 (m, 2 H, H-4^{Gal}, H-4^{Glc}), 3.78–3.74 (m, 1 H, H-5^{Gal}), 3.59 (dd, 1 H, $J_{3,4}$ = 2.7 Hz, $J_{2,3}$ = 9.9 Hz, H-3^{Gal}), 3.54–3.34 (m, 7 H, H-6a^{Gal}, H-2^{Glc}, H-6a^{Glc}, H-6b^{Glc}, H-1^{Ins}, H-3^{Ins}, H-5^{Ins}), 3.27 (dd, 1 H, $J_{5,6b}$ = 4.5 Hz, J_{gem} = 9.7 Hz, H-6b^{Gal}), 2.88 (br s, 1 H, 6-OH^{Glc}), 2.69 (d, 1 H, $J_{2,OH}$ = 8.3 Hz, 2-OH^{Gal}); ^{13}C NMR (125 MHz, $CDCl_3$) δ 139.2, 139.0, 138.8, 138.7, 138.5, 138.5, 138.3, 138.2, 137.6, 135.2, 133.4, 133.3, 128.6, 128.5, 128.5, 128.5, 128.4, 128.4, 128.3, 128.3, 128.2, 128.2, 128.2, 128.1, 128.1, 128.0, 127.9, 127.8, 127.8, 127.7, 127.7, 127.7, 127.6, 127.6, 127.5, 127.4, 127.4, 127.3, 127.1, 126.4, 126.4, 126.3, 101.1 (C-1^{Gal}), 96.6 (C-1^{Glc}), 84.1, 81.7, 81.6, 81.3, 80.2, 80.1, 79.7, 79.2, 76.2, 75.8, 75.6, 75.5, 74.8, 74.7, 74.5, 74.0, 73.7, 73.6, 73.0, 72.7, 72.6, 71.7, 71.4, 71.3, 70.8, 69.8, 69.5, 60.8; HRMS (ESI) *m/z*: found $[M+Na]^+$ 1477.6353, $C_{92}H_{94}O_{16}$ calcd for $[M+Na]^+$ 1477.6434.

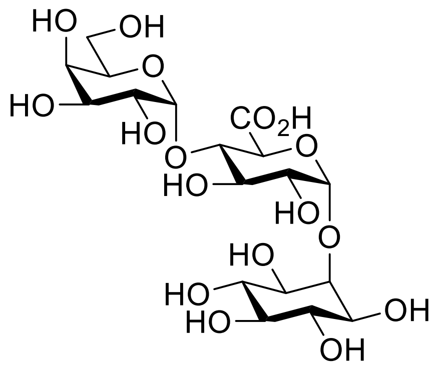

α-D-Galactopyranosyl-(1→4)-α-D-glucopyranosyluronic acid-(1→2)-*myo*-inositol (2)

To a solution of **23** (34.4 mg, 23.6 μmol) in CH_2Cl_2 (2.3 mL) were added TEMPO (4.1 mg, 26 μmol), H_2O (840 μL), and iodobenzene diacetate (8.9 mg, 27.5 μmol) at room temperature and the reaction mixture was stirred at the same temperature. The progress of the reaction was monitored by TLC (*n*-hexane/acetone = 2:1). After 17 h of stirring, iodobenzene diacetate (15.2 mg, 47.2 μmol) was added. After 6 h of stirring, iodobenzene diacetate (15.2 mg, 47.2 μmol) was added. An additional 1 h of stirring was followed by the addition of TEMPO (4.1 mg, 26 μmol) and iodobenzene diacetate (15.2 mg, 47.2 μmol). After 11 h stirring, iodobenzene diacetate (15.2 mg, 47.2 μmol) was added. The reaction mixture was stirred for an additional 3 h and then concentrated in vacuo. The resulting residue was exposed to high vacuum for 2 h and dissolved in DMF (470 μL). To the solution were added benzyl bromide (11.2 μL, 94.4 μmol) and $NaHCO_3$ (15.9 mg, 18.9 μmol) at room temperature and the reaction mixture was stirred at the same temperature. The progress of the reaction was monitored by TLC (*n*-hexane/acetone = 2:1). After 28 h of stirring, the reaction mixture was diluted with *n*-hexane/EtOAc (4:1) and washed with sat. aq. $NaHCO_3$. The organic layer was dried over Na_2SO_4, filtered off, and concentrated in vacuo. The resulting residue was purified using flash silica gel column chromatography (*n*-hexane/acetone = 85:15) and size-exclusion chromatography on Sephadex LH-20 ($CHCl_3$/MeOH = 1:1) to yield the corresponding benzyl ester **24** (11.5 mg, 31%, 2 steps) containing trace impurities as a colorless syrup. The formation of **24** was confirmed by ^{1}H NMR spectroscopy {^{1}H NMR (500 MHz, $CDCl_3$) δ 7.86–6.86 (m, 57 H, Ar), 5.65 (d, 1 H, $J_{1,2}$ = 3.6 Hz, H-1^{GlcA}), 5.46 (d, 1 H, $J_{1,2}$ = 3.9 Hz, H-1^{Gal}), 5.13–4.30 (m, 24 H, H-5^{GlcA}, H-2^{Ins}, 22 ArCH_2), 4.14–3.96 (m, 5 H, H-2^{Gal}, H-3^{GlcA}, H-4^{GlcA}, H-4^{Ins}, H-6^{Ins}), 3.92 (br d, 1 H, $J_{3,4}$ = 1.4 Hz, H-4^{Gal}), 3.79–3.73 (m, 1 H, H-6a^{Gal}), 3.62–3.55 (m, 2 H, H-2^{GlcA}, H-6b^{Gal}), 3.51–3.45 (m, 2 H, H-3^{Gal}, H-5^{Gal}), 3.42 (t, 1 H, $J_{4,5}$ = $J_{5,6}$ = 9.3 Hz, H-5^{Ins}), 3.33 (dd, 1 H, $J_{1,2}$ = 1.5 Hz, $J_{1,6}$ = 9.9 Hz, H-1^{Ins}), 3.28 (dd, 1 H, $J_{2,3}$ = 2.3 Hz, $J_{3,4}$ = 9.9 Hz, H-3^{Ins}), 2.53 (d, 1 H, $J_{2,OH}$ = 8.3 Hz, 2-OH^{Gal})} and mass spectroscopy {HRMS (ESI) *m*/*z*: found $[M+Na]^+$ 1581.6694, $C_{99}H_{98}O_{17}$ calcd for $[M+Na]^+$ 1581.6696}. The obtained benzyl ester **24** (11.5 mg, 7.37 μmol) was exposed to high vacuum overnight and then dissolved in

MeOH/THF (2:1, 2.4 mL). To the solution was added Pd/C (10% on carbon: 15.7 mg, 14.7 μmol) at room temperature. After degassing and filling with H_2 gas three times, the reaction mixture was stirred at room temperature under H_2 gas atmosphere. The progress of the reaction was monitored by TLC ($CHCl_3$/MeOH/AcOH = 1:3:0.1). After 15 h of stirring, the reaction mixture was filtered through a pad of Celite® and the pad was rinsed with MeOH. The combined filtrate was concentrated in vacuo. The resulting residue was purified using size-exclusion chromatography on Sephadex LH-20 (MeOH/H_2O = 1:1) and lyophilized to yield **1** (3.6 mg, 95%) as a white powder.

^{1}H NMR (600 MHz, D_2O) δ 5.46 (d, 1 H, $J_{1,2}$ = 3.7 Hz, H-1^{Gal}), 5.14 (d, 1 H, $J_{1,2}$ = 3.6 Hz, H-1^{GlcA}), 4.31 (d, 1 H, $J_{4,5}$ = 10.0 Hz, H-5^{GlcA}), 4.06 (br s, 1 H, H-2^{Ins}), 3.96–3.91 (m, 3 H, H-3^{GlcA}, H-4^{Gal}, H-6a^{Gal}), 3.79 (dd, 1 H, $J_{3,4}$ = 2.6 Hz, $J_{2,3}$ = 10.4 Hz, H-3^{Gal}), 3.76–3.59 (m, 7 H, H-2^{Gal}, H-5^{Gal}, H-6b^{Gal}, H-2^{GlcA}, H-4^{GlcA}, H-4^{Ins}, H-6^{Ins}), 3.58–3.50 (m, 2 H, H-1^{Ins}, H-3^{Ins}), 3.24 (t, 1 H, $J_{4,5}$ = $J_{5,6}$ = 9.3 Hz, H-5^{Ins}); ^{13}C NMR (125 MHz, D_2O) δ 176.2, 100.4, 98.4, 81.4, 76.6, 74.5, 73.6, 73.0, 72.7, 71.9, 70.6, 70.4, 69.3, 69.0, 68.6, 60.8; HRMS (ESI) *m/z*: found [M−H]$^-$ 517.1408, $C_{18}H_{29}O_{17}$ calcd for [M−H]$^-$ 517.1410.

V. Synthesis of Man-type inositol glycan **3**

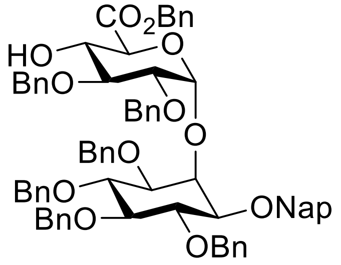

Benzyl 2,3-di-*O*-benzyl-α-D-glucopyranosyluronate-(1→2)-1D-3,4,5,6-tetra-*O*-benzyl-1-*O*-(2-naphthalenylmethyl)-*myo*-inositol (25)

To a solution of **17** (300 mg, 293 μmol) in CH_2Cl_2 (28.6 mL) were added TEMPO (50.5 mg, 323 μmol), H_2O (10.4 mL), and iodobenzene diacetate (189 mg, 586 μmol) at room temperature and the reaction mixture was stirred at the same temperature. The progress of the reaction was monitored by TLC (*n*-hexane/acetone = 2:1). After 17 h of stirring, iodobenzene diacetate (47.2 mg, 147 μmol) was added. After 3 h of stirring, iodobenzene diacetate (94.3 mg, 293 μmol) was added. The reaction mixture was stirred for an additional 1 h and then concentrated in vacuo. The resulting residue was exposed to high vacuum for 2 h and dissolved in DMF (5.9 mL). To the solution were added benzyl bromide (139 μL, 1.17 mmol) and $NaHCO_3$ (197 mg, 2.34 mmol) at room temperature and the reaction mixture was stirred at the same temperature. The progress of the reaction was monitored by TLC (*n*-

hexane/acetone = 2:1). After 17.5 h of stirring, the reaction mixture was diluted with *n*-hexane/EtOAc (4:1) and washed with sat. aq. $NaHCO_3$. The organic layer was dried over Na_2SO_4, filtered off, and concentrated in vacuo. The resulting residue was purified using flash silica gel column chromatography (toluene/acetone = 99:1) to yield **25** (180 mg, 55%, 2 steps) as a white powder.

$[\alpha]_D^{20}$ +34.2° (c 0.8, $CHCl_3$); ^1H NMR (500 MHz, $CDCl_3$) δ 7.86–6.87 (m, 42 H, Ar), 5.67 (d, 1 H, $J_{1,2}$ = 3.5 Hz, H-1^{GlcA}), 5.08–4.47 (m, 16 H, H-5^{GlcA}, 15 ArCH_2), 4.43 (br t, 1 H, $J_{1,2}$ = $J_{2,3}$ = 1.9 Hz, H-2^{Ins}), 4.38 (d, 1 H, J = 12.2 Hz, ArCH_2), 4.15 (t, 1 H, $J_{1,6}$ = $J_{5,6}$ = 9.6 Hz, H-6^{Ins}), 3.99 (t, 1 H, $J_{3,4}$ = $J_{4,5}$ = 9.6 Hz, H-4^{Ins}), 3.87 (t, 1 H, $J_{2,3}$ = $J_{3,4}$ = 9.0 Hz, H-3^{GlcA}), 3.78–3.72 (m, 1 H, H-4^{GlcA}), 3.51 (dd, 1 H, H-2^{GlcA}), 3.45 (t, 1 H, H-5^{Ins}), 3.39 (dd, 1 H, H-1^{Ins}), 3.33 (dd, 1 H, H-3^{Ins}), 2.89 (d, 1 H, $J_{4,OH}$ = 2.4 Hz, 4-OH^{GlcA}); ^{13}C NMR (125 MHz, $CDCl_3$) δ 170.8, 139.0, 138.7, 138.7, 138.5, 138.1, 137.9, 135.1, 135.0, 133.2, 133.1, 128.6, 128.4, 128.4, 128.3, 128.3, 128.3, 128.2, 128.1, 128.0, 127.7, 127.6, 127.5, 127.4, 127.4, 127.3, 127.2, 127.1, 126.9, 126.3, 126.3, 126.2, 97.2, 83.7, 81.4, 81.3, 81.0, 79.7, 78.3, 76.2, 75.7, 75.4, 75.3, 74.0, 73.0, 72.0, 72.0, 71.3, 70.7, 67.2; HRMS (ESI) *m/z*: found $[M+Na]^+$ 1149.4761, $C_{72}H_{70}O_{12}$ calcd for $[M+Na]^+$ 1149.4759.

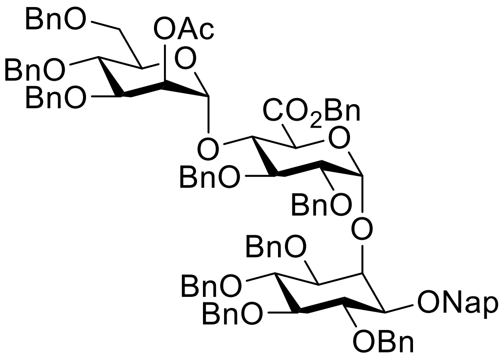

2-*O*-Acetyl-3,4,6-tri-*O*-benzyl-α-D-mannopyranosyl-(1→4)-benzyl 2,3-di-*O*-benzyl-α-D-glucopyranosyluronate-(1→2)-1D-3,4,5,6-tetra-*O*-benzyl-1-*O*-(2-naphthalenylmethyl)-*myo*-inositol (27)

25 (64.3 mg, 57.0 μmol) and 2-*O*-acetyl-3,4,6-tri-*O*-benzyl-D-mannopyranosyl 2,2,2-trichloroacetimidate **26**[11] (α/β = >20:1, 54.5 mg, 85.6 μmol) were mixed in a pear-shaped flask, and then residual H_2O was azeotropically removed with dry toluene. After exposure to high vacuum overnight, the mixture was dissolved in CH_2Cl_2 (739 μL) and the solution was then transferred to a two-necked flask containing pre-activated molecular sieves AW-300 (95.0 mg) via cannula at room temperature. After 15 min of stirring at −20 °C, TMSOTf (0.18 M solution in CH_2Cl_2: 47.5 μL, 8.56 μmol) was added to the mixture and the reaction mixture was stirred at the same temperature. The progress of the reaction was monitored by TLC (*n*-

hexane/EtOAc = 3:1). After 15 min of stirring, the reaction mixture was filtered through a pad of Celite®, which was rinsed with $CHCl_3$. The combined filtrate was washed with sat. aq. $NaHCO_3$ and brine. The organic layer was dried over Na_2SO_4, filtered off, and concentrated in vacuo. The resulting residue was purified using size-exclusion chromatography on Sephadex LH-20 ($CHCl_3$/MeOH = 1:1) and flash silica gel column chromatography (*n*-hexane/EtOAc = 80:20) to yield **27** (83 mg, 91%, α only) as a colorless syrup. The newly formed α-glycosidic linkage was confirmed by the $^1J_{C1,H1}$ coupling constant of 175 Hz.[13]

$[\alpha]_D^{20}$ +23.0° (c 0.9, $CHCl_3$); ^{1}H NMR (500 MHz, $CDCl_3$) δ 7.87–6.84 (m, 57 H, Ar), 5.63 (d, 1 H, $J_{1,2}$ = 3.6 Hz, H-1^{GlcA}), 5.54–5.49 (m, 2 H, H-1^{Man}, H-2^{Man}), 5.11–4.34 (m, 22 H, H-5^{GlcA}, 21 ArCH_2), 4.29 (br s, 1 H, H-2^{Ins}), 4.25 (d, 1 H, J_{gem} = 10.7 Hz, ArCH_2), 4.11 (t, 1 H, $J_{3,4}$ = $J_{4,5}$ = 9.6 Hz, H-4^{Ins}), 4.06 (t, 1 H, $J_{2,3}$ = $J_{3,4}$ = 9.5 Hz, H-4^{GlcA}), 4.04–3.95 (m, 2 H, H-3^{GlcA}, H-6^{Ins}), 3.93 (t, 1 H, $J_{3,4}$ = $J_{4,5}$ = 9.6 Hz, H-4^{Man}), 3.86–3.80 (m, 2 H, H-3^{Man}, H-6a^{Man}), 3.68–3.53 (m, 3 H, H-5^{Man}, H-6b^{Man}, H-2^{GlcA}), 3.41 (t, 1 H, $J_{5,6}$ = 9.3 Hz, H-5^{Ins}), 3.31 (dd, 1 H, $J_{2,3}$ = 1.6 Hz, H-3^{Ins}), 3.24 (dd, 1 H, $J_{1,2}$ = 2.2 Hz, $J_{1,6}$ = 9.9 Hz, H-1^{Ins}), 2.00 (s, 3 H, Ac); ^{13}C NMR (125 MHz, $CDCl_3$) δ 170.4, 170.0, 139.0, 138.7, 138.5, 138.4, 138.2, 138.0, 137.9, 137.9, 135.0, 134.6, 133.2, 133.1, 128.5, 128.4, 128.3, 128.3, 128.3, 128.2, 128.2, 128.1, 128.0, 128.0, 127.9, 127.9, 127.8, 127.7, 127.7, 127.6, 127.5, 127.4, 127.3, 127.3, 127.1, 126.9, 126.3, 126.3, 126.2, 98.4 (C-1^{Man}), 97.2 (C-1^{GlcA}), 83.7, 81.4, 81.0, 80.8, 80.5, 79.1, 78.4, 77.2, 76.1, 75.4, 75.3, 75.2, 75.1, 74.6, 73.9, 73.8, 73.5, 73.4, 72.0, 71.9, 71.8, 71.2, 70.3, 68.4, 68.3, 67.6, 21.0; HRMS (ESI) *m/z*: found $[M+Na]^+$ 1623.6797, $C_{101}H_{100}O_{18}$ calcd for $[M+Na]^+$ 1623.6802.

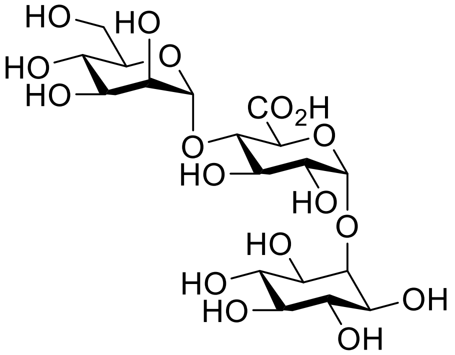

α-D-Mannopyranosyl-(1→4)-α-D-glucopyranosyluronic acid-(1→2)-*myo*-inositol (3)

To a solution of **27** (31.9 mg, 19.9 μmol) in MeOH/THF (3:1, 6.8 mL) was added Pd/C (10% on carbon: 42.4 mg, 39.8 μmol) at room temperature. After degassing and filling with H_2 gas three times, the reaction mixture was stirred at room temperature under H_2 gas atmosphere. The progress of the reaction was monitored by TLC ($CHCl_3$/MeOH/AcOH = 1:3:0.1). After 24 h of stirring, the reaction mixture was filtered through a pad of Celite® and the pad was rinsed

with MeOH. The combined filtrate was concentrated in vacuo. The resulting residue was dissolved in 1 M aq. NaOH (1.0 mL, 1.0 mmol) and the reaction mixture was stirred at room temperature and the reaction mixture was stirred at the same temperature. The progress of the reaction was monitored by TLC ($CHCl_3$/MeOH/AcOH = 1:3:0.1). After 9 h of stirring, the reaction was neutralized with Muromac® (H^+ form) at 0 °C. The resulting mixture was filtered off and then concentrated in vacuo. The resulting residue was purified using size-exclusion chromatography on Sephadex LH-20 (MeOH/H_2O = 1:1) and lyophilized to yield **3** (9.5 mg, 92%, 2 steps) as a white powder. The physical characteristics of **3** were consistent with the reported values.[14]

5. References

[1] M. Islam, R. Y. Hasi, Y. Umemura, H.-N. Tanaka, Y. Kondo, T. Ishikawa, M. Nagano, H. Ali, R. Kawakami, M. Aihara, T. Tanaka, *J. Biochem.* **2025**, *177*, 387–394.

[2] R. Y. Hasi, D. Majima, K. Morito, H. Ali, K. Kogure, M. Nanjundan, J. Hayashi, R. Kawakami, K. Kanemaru, T. Tanaka, *J. Chromatogr. B* **2020**, *1152*, 122213.

[3] R. Y. Hasi, T. Ishikawa, K. Sunagawa, Y. Takai, H. Ali, J. Hayashi, R. Kawakami, K. Yuasa, M. Aihara, K. Kanemaru, H. Imai, T. Tanaka, *FEBS Lett.* **2022**, *596*, 3024–3036.

[4] S. Yasuno, T. Murata, K. Kokubo, T. Yamaguchi, M. Kamei, *Biosci. Biotech. Biochem.* **1997**, *61*, 1944–1946.

[5] C. Kadooka, Y. Tanaka, R. Kishida, D. Hira, T. Oka, *mSphere* **2024**, *9*, e00100-24.

[6] F. F. J. de Kleijne, S. J. Moons, P. B. White, T. J. Boltje, *Org. Biomol. Chem.* **2020**, *18*, 1165–1184.

[7] V. Gannedi, A. Ali, P. P. Singh, R. A. Vishwakarma, *J. Org. Chem.* **2020**, *85*, 7757–7771.

[8] D. Crich, M. de la Mora, A. U. Vinod, *J. Org. Chem.* **2003**, *68*, 8142–8148.

[9] R. L. Halcomb, S. J. Danishefsky, *J. Am. Chem. Soc.* **1989**, *111*, 6661–6666.

[10] L. Alberch, G. Cheng, S.-K. Seo, X. Li, F. P. Boulineau, A. Wei, *J. Org. Chem.* **2011**, *76*, 2532–2547.

[11] F. Yamazaki, S. Sato, T. Nukada, Y. Ito, T. Ogawa, *Carbohydr. Res.* **1990**, *201*, 31–50.

[12] D. F. Taber, P. W. DeMatteo, R. A. Hassan, *Org. Synth.* **2013**, *90*, 350–357.

[13] J. Ø. Duus, C. H. Gotfredsen, K. Bock, *Chem. Rev.* **2000**, *100*, 4589–4614.

[14] C. K. Smith, C. M. Hewage, S. C. Fry, I. H. Sadler, *Phytochemistry* **1999**, *52*, 387–396.

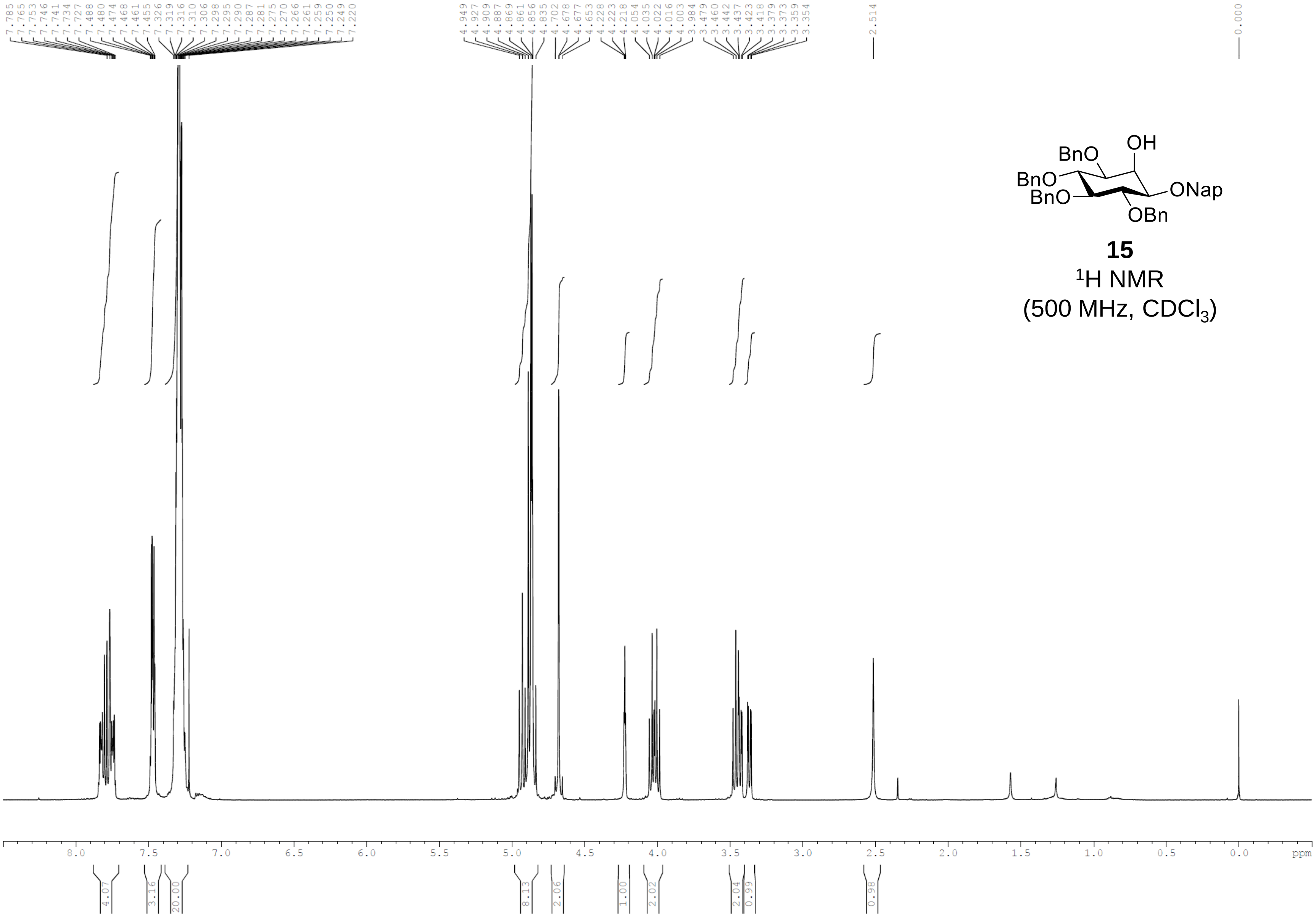

15

^{1}H NMR

(500 MHz, $CDCl_3$)

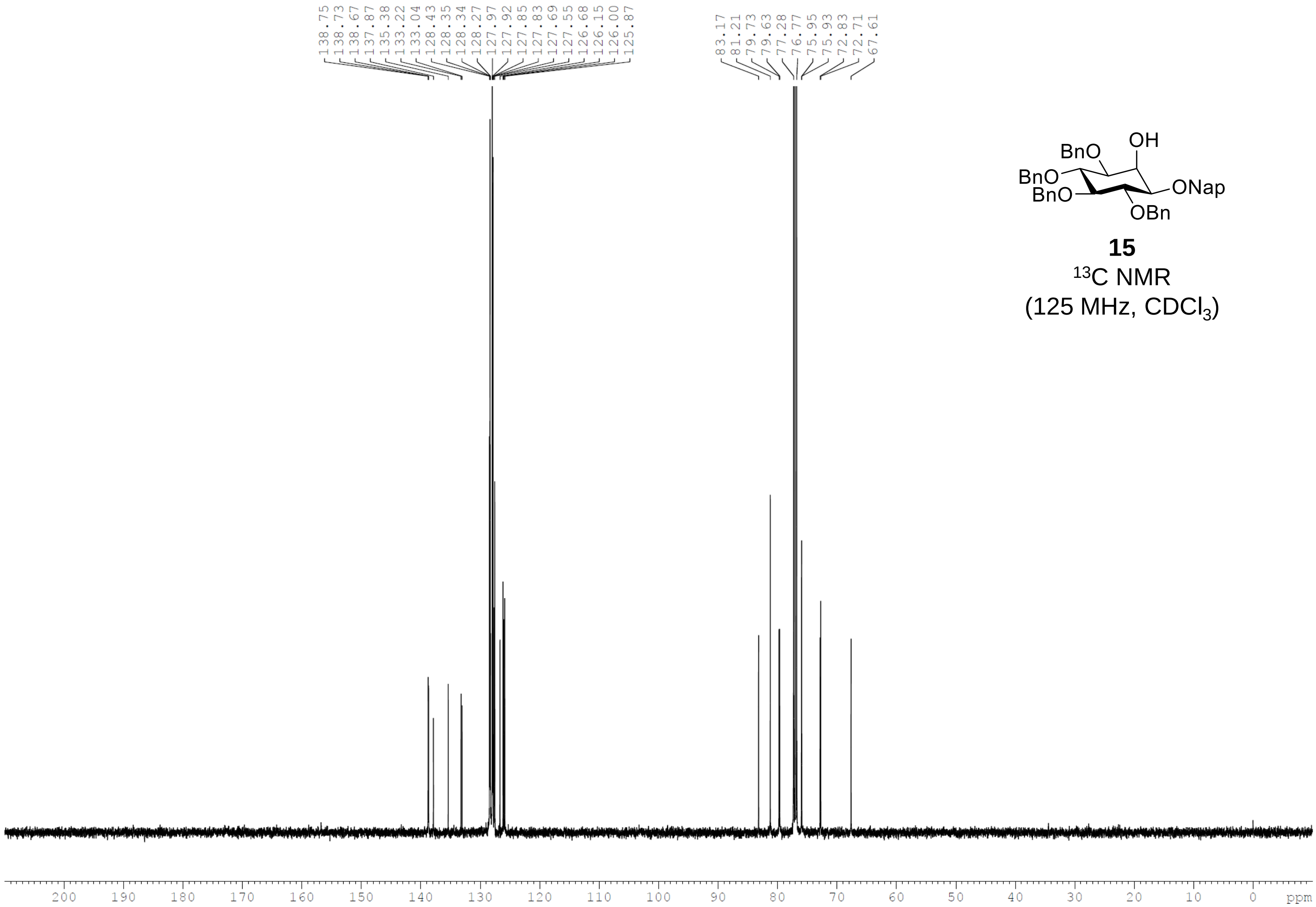

15

^{13}C NMR

(125 MHz, $CDCl_3$)

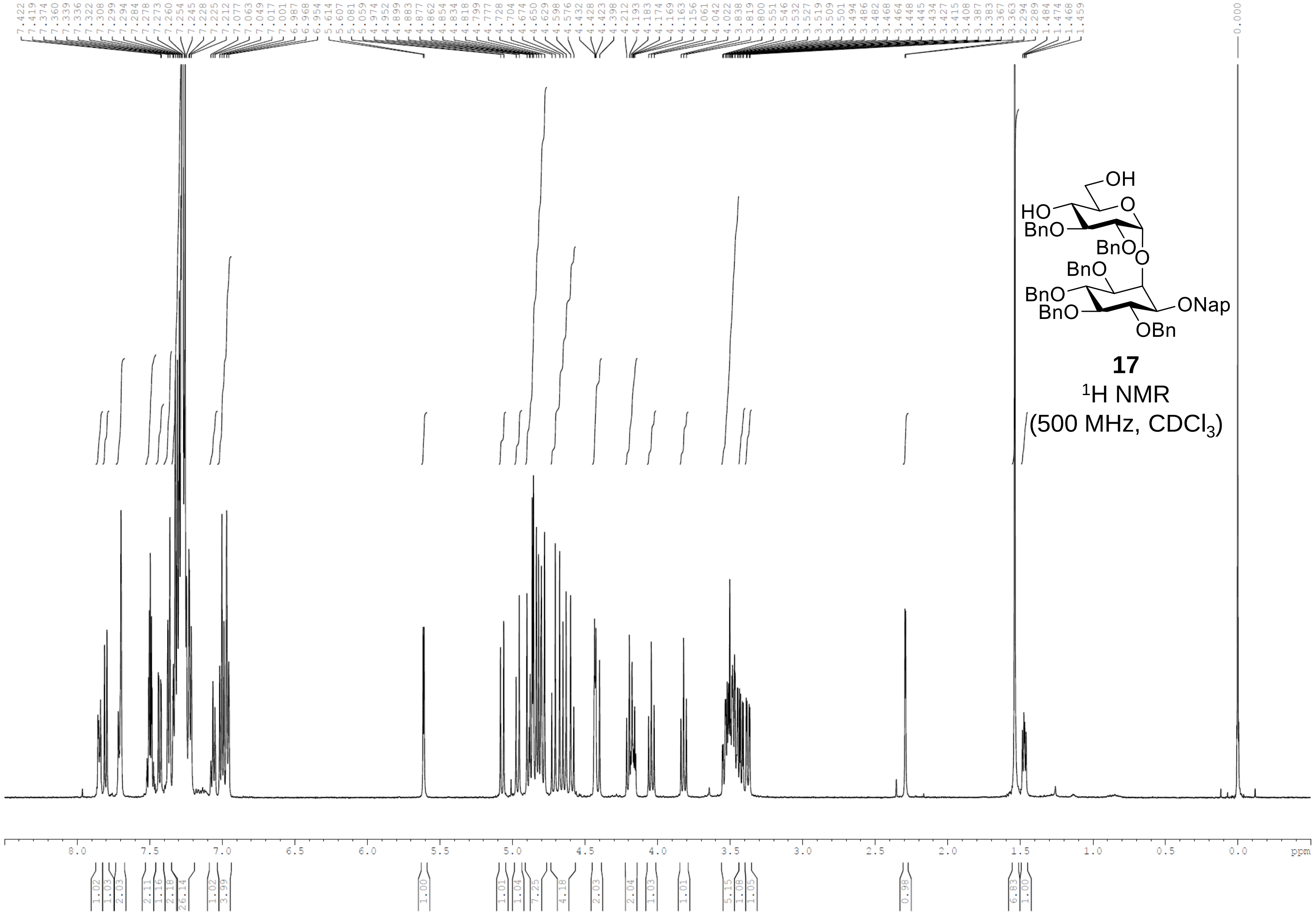

17

^{1}H NMR

(500 MHz, $CDCl_3$)

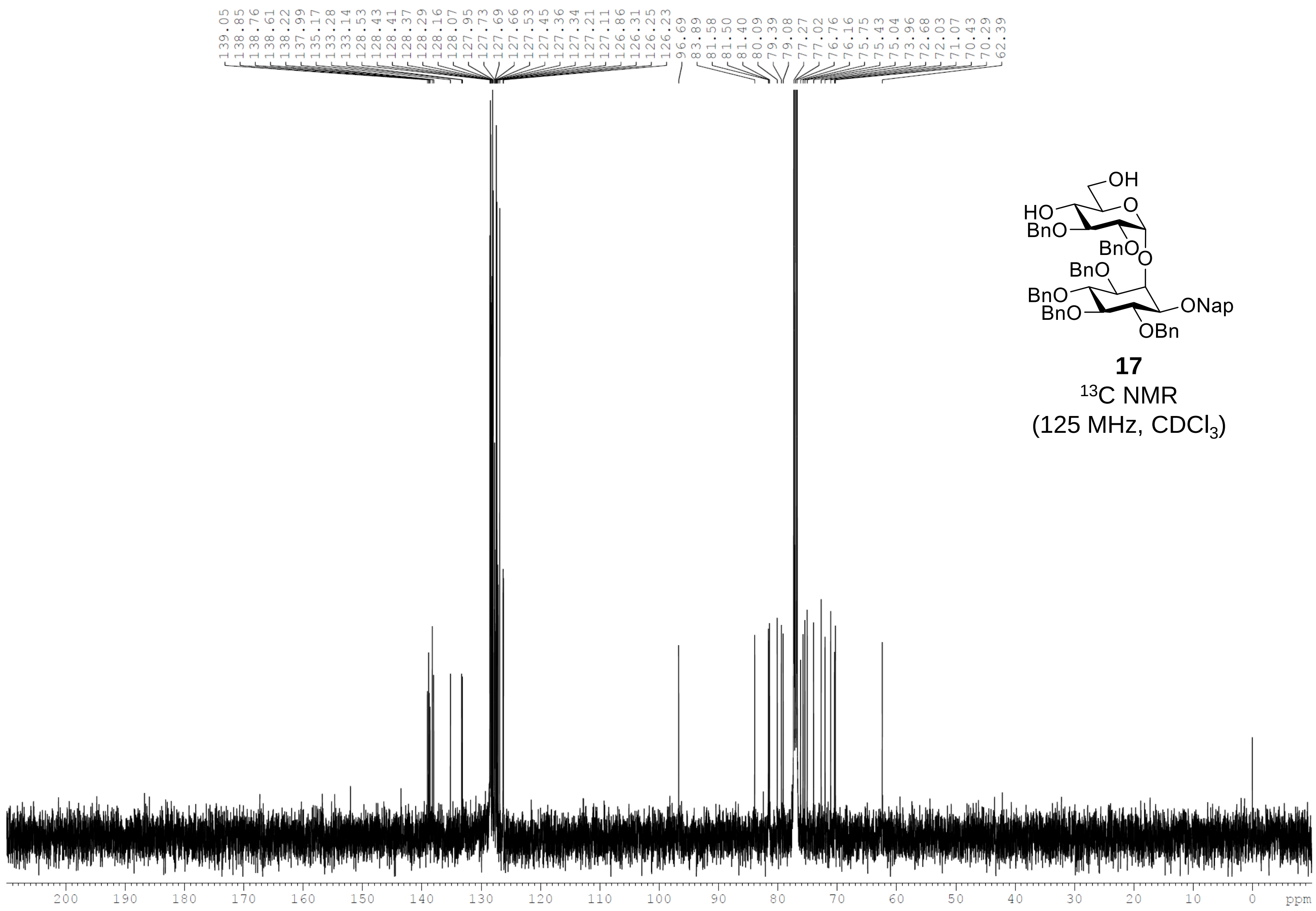
139.05
138.85
138.76
138.61
138.22
137.99
135.17
133.28
133.14
128.53
128.43
128.41
128.37
128.29
128.16
128.07
127.95
127.73
127.69
127.66
127.53
127.45
127.36
127.34
127.21
127.11
126.86
126.31
126.25
126.23
96.69
83.89
81.58
81.50
81.40
80.09
79.39
79.08
77.27
77.02
76.76
76.16
75.75
75.43
75.04
73.96
72.68
72.03
71.07
70.43
70.29
62.39
OH
O
HO
BnO
BnO
O
BnO
BnO
BnO
ONap
OBn
17
13C NMR
(125 MHz, CDCl3)
200 190 180 170 160 150 140 130 120 110 100 90 80 70 60 50 40 30 20 10 0 ppm

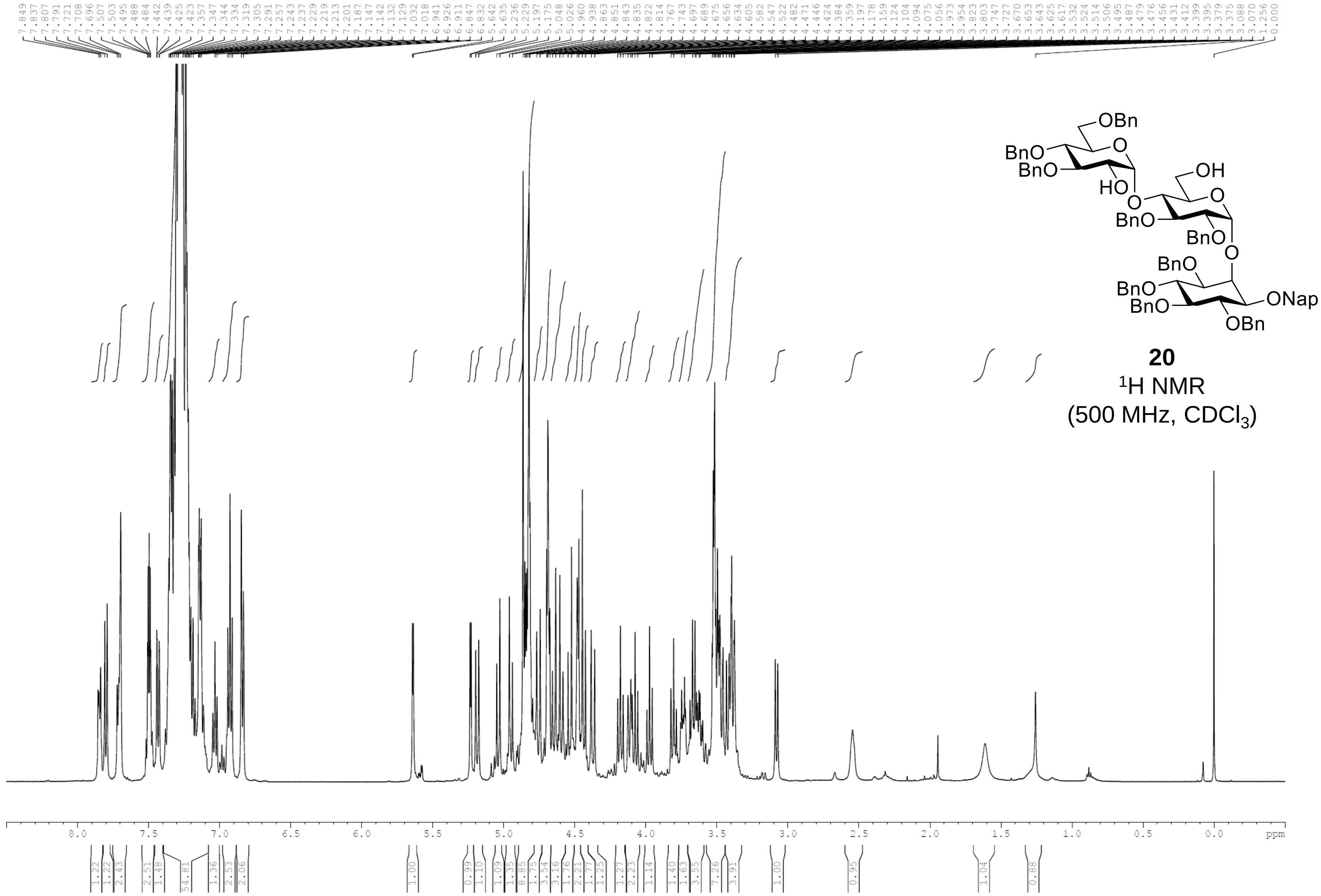

OBn
BnO
BnO
HO
O
OH
BnO
BnO
BnO
ONap
OBn
20
^{1}H NMR
(500 MHz, $CDCl_3$)
ppm

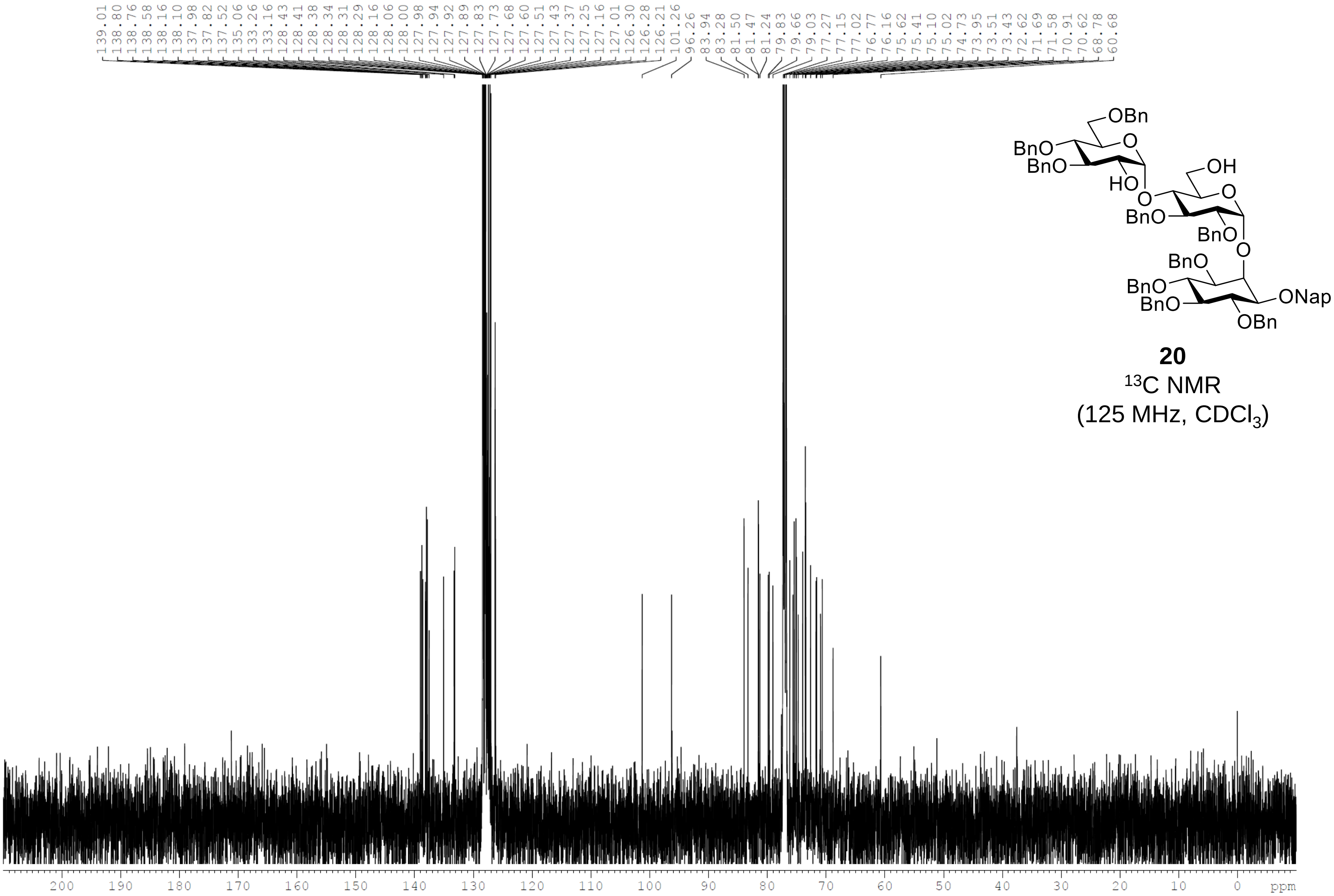

20

^{13}C NMR

(125 MHz, $CDCl_3$)

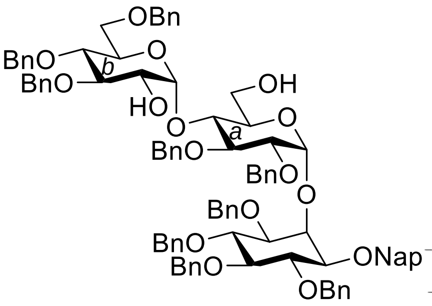

20

HMQC

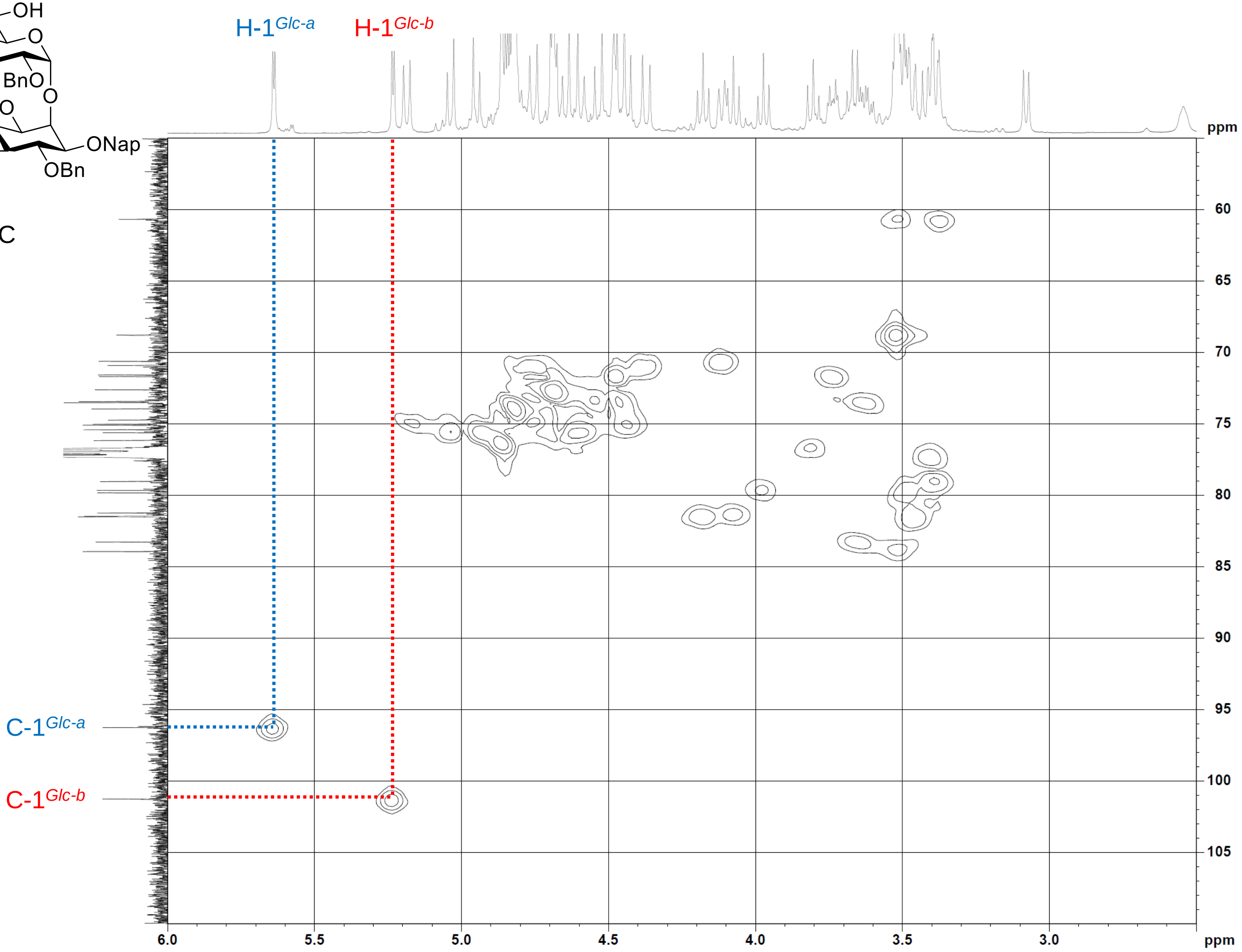

H-1Glc-a
H-1Glc-b
C-1Glc-a
C-1Glc-b
ppm
60
65
70
75
80
85
90
95
100
105
6.0
5.5
5.0
4.5
4.0
3.5
3.0
ppm

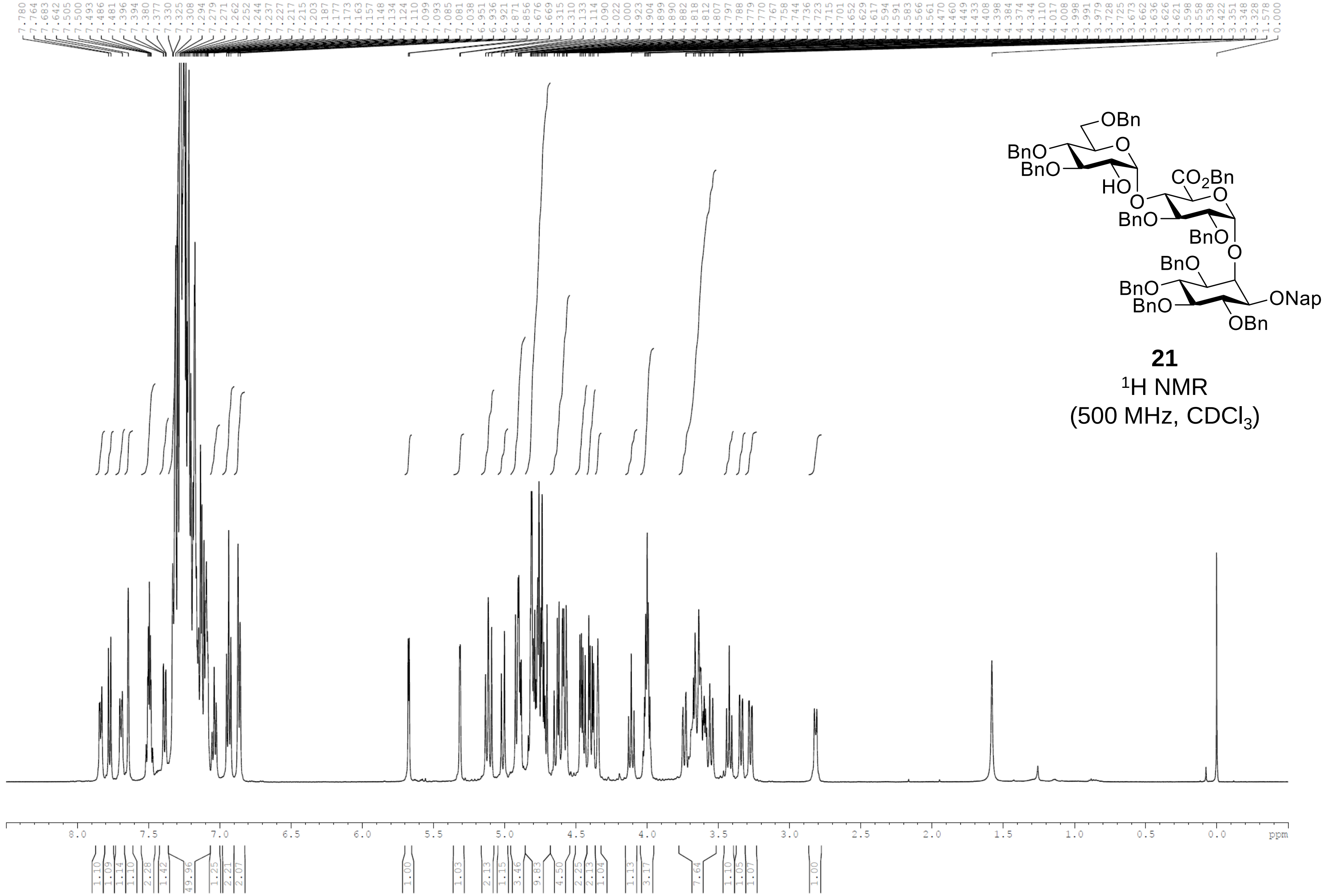

21
^{1}H NMR
(500 MHz, $CDCl_3$)

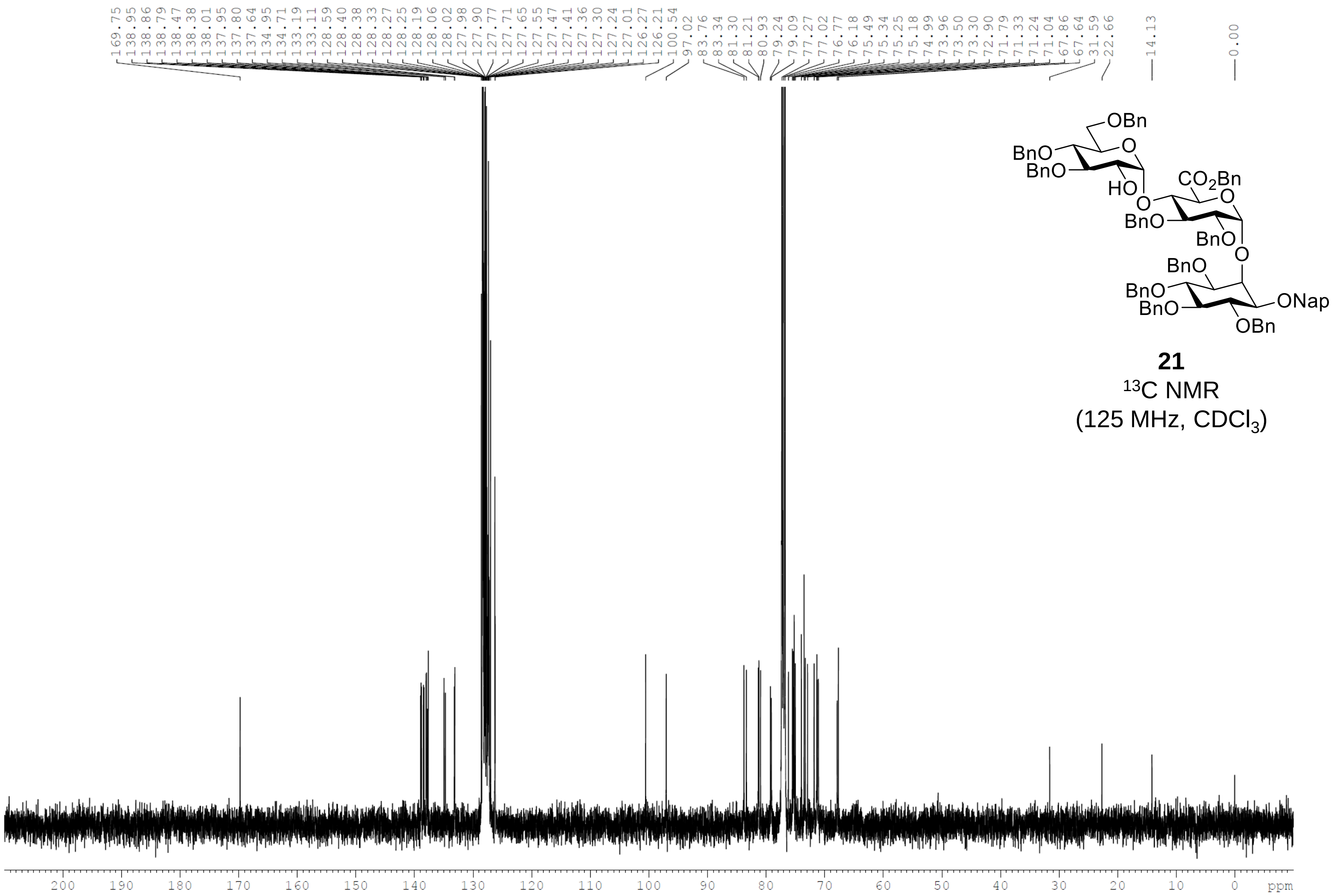

21

^{13}C NMR

(125 MHz, $CDCl_3$)

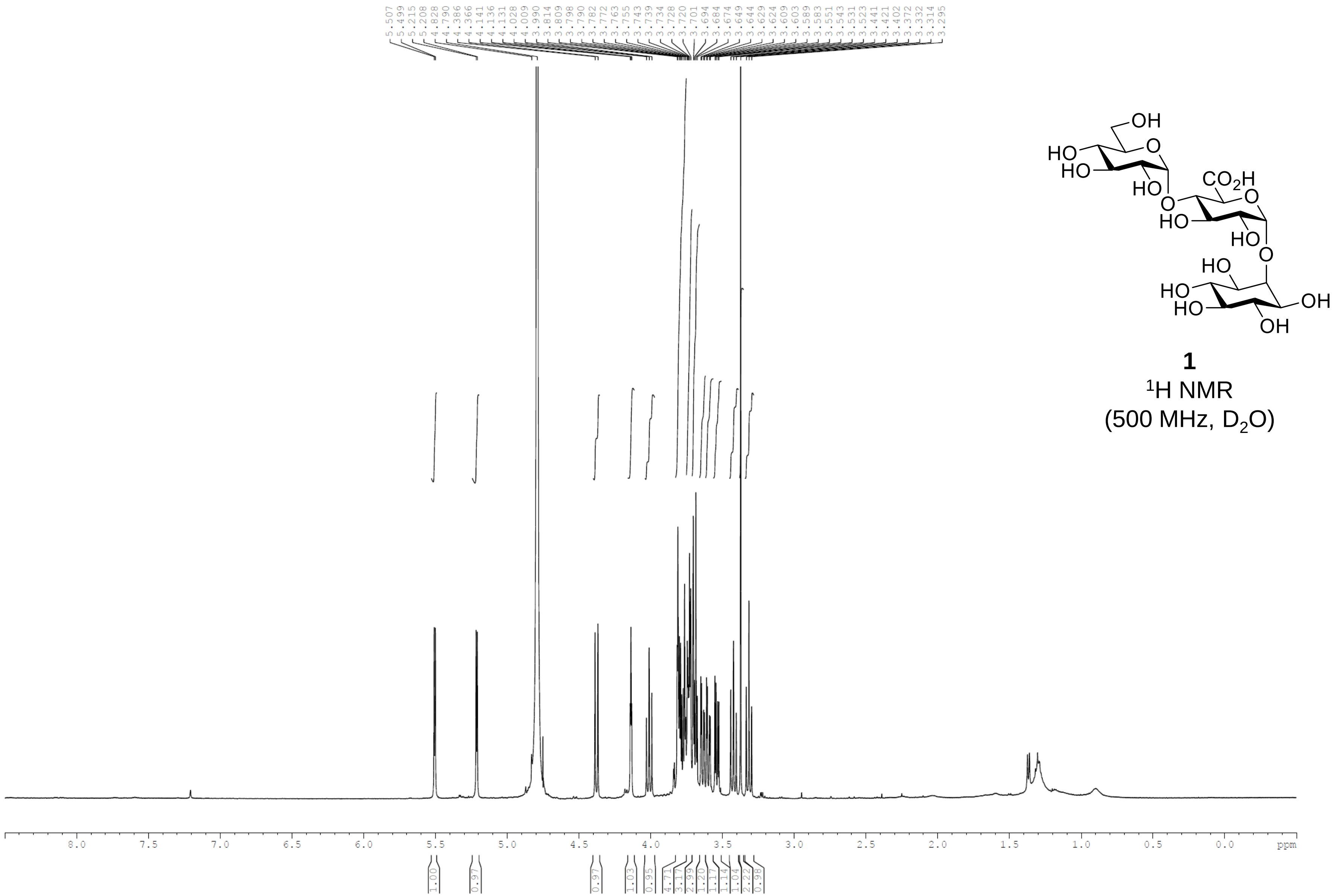

1

^{1}H NMR

(500 MHz, D_2O)

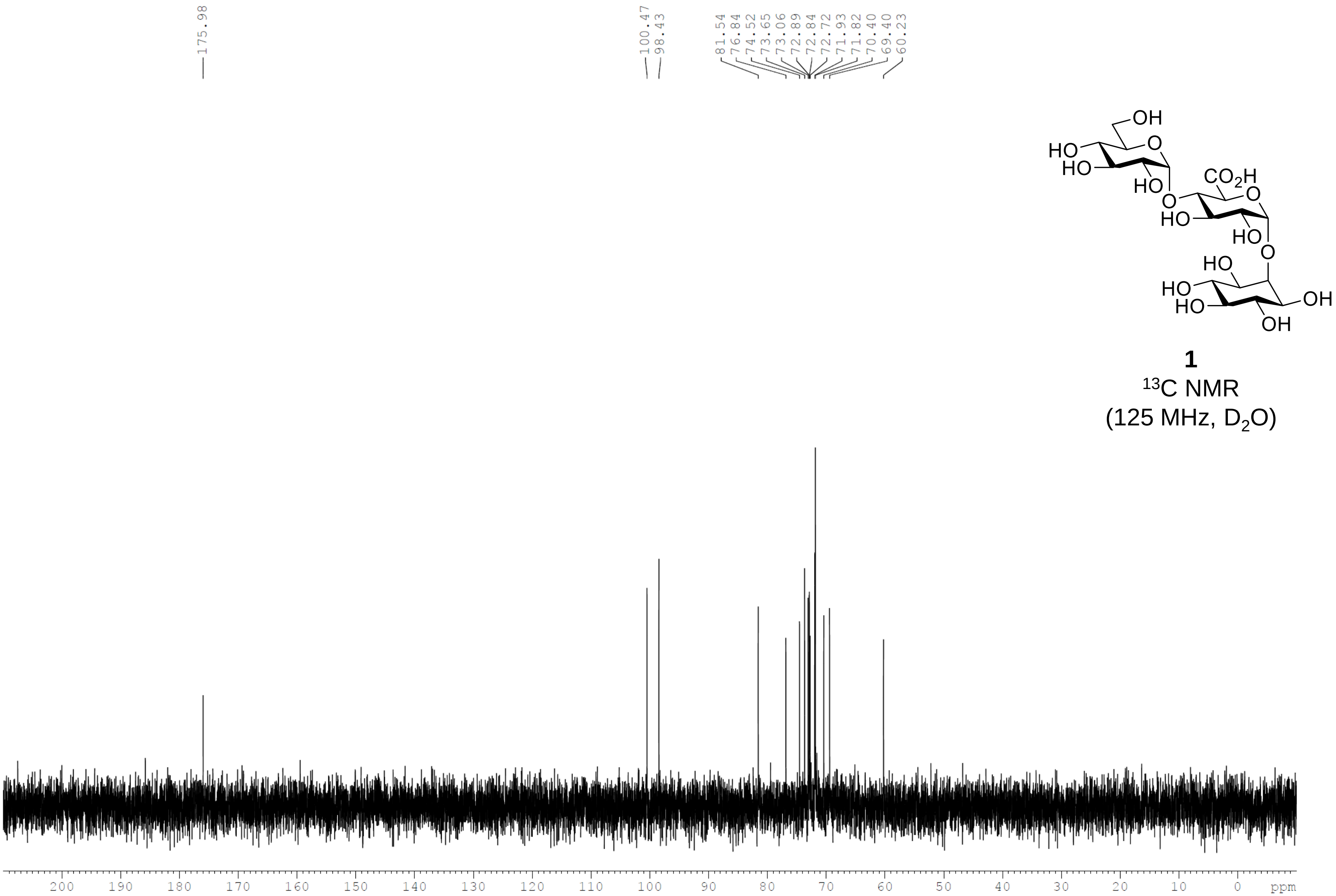

1

^{13}C NMR

(125 MHz, D_2O)

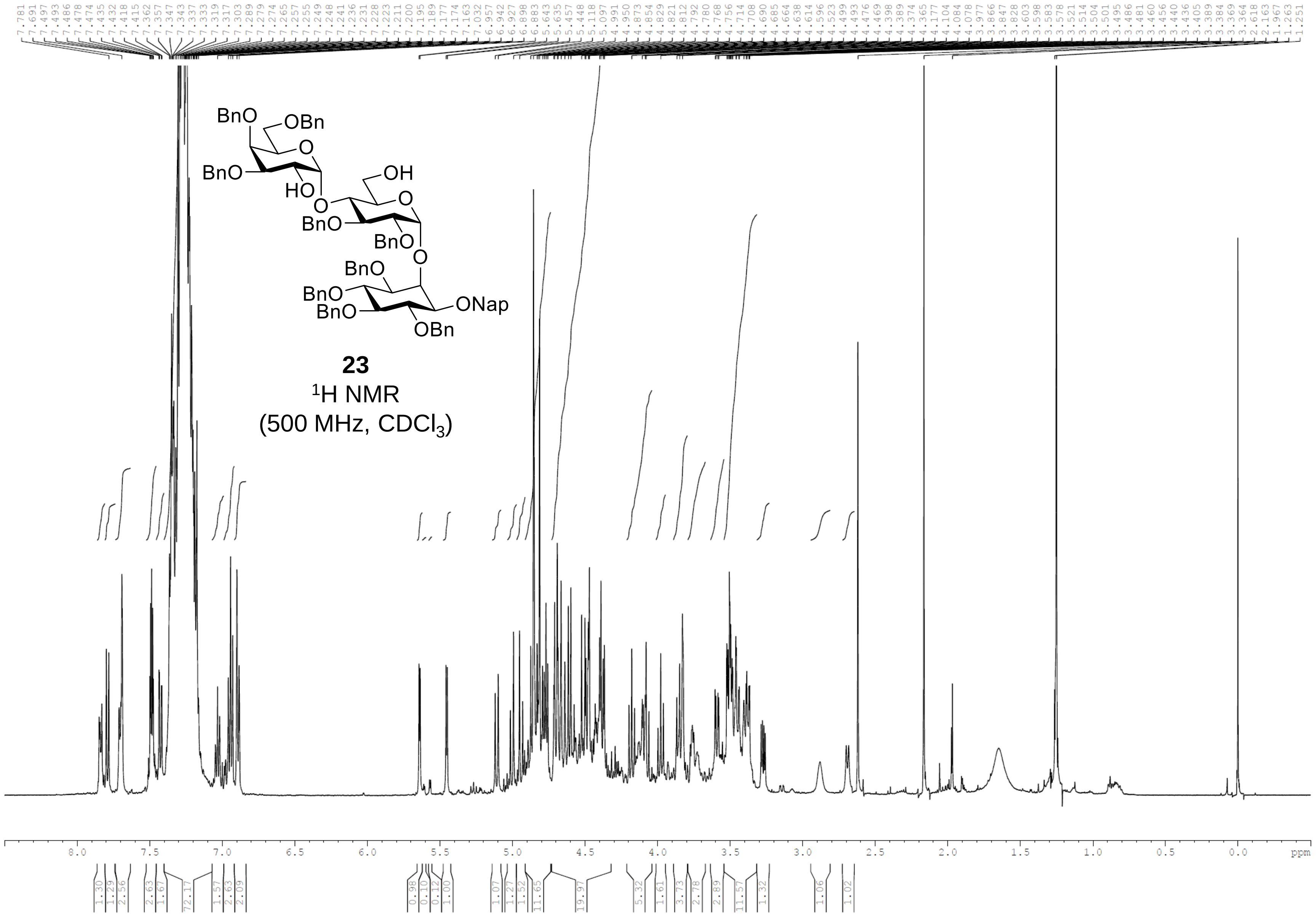

23

^{1}H NMR

(500 MHz, $CDCl_3$)

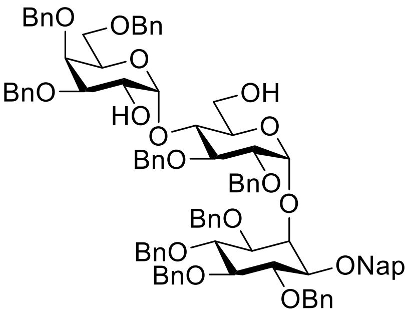

23
^{13}C NMR
(125 MHz, $CDCl_3$)

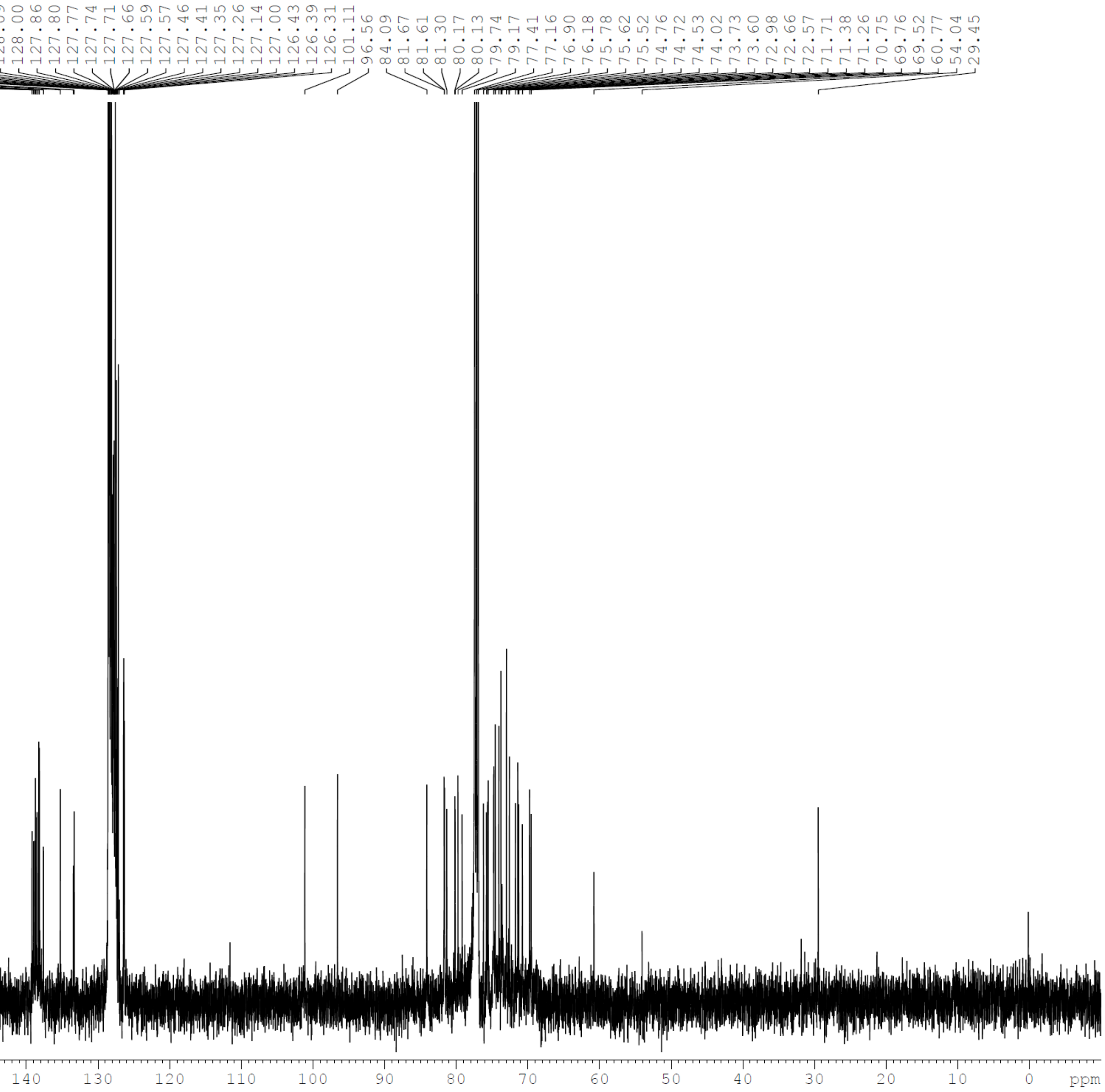

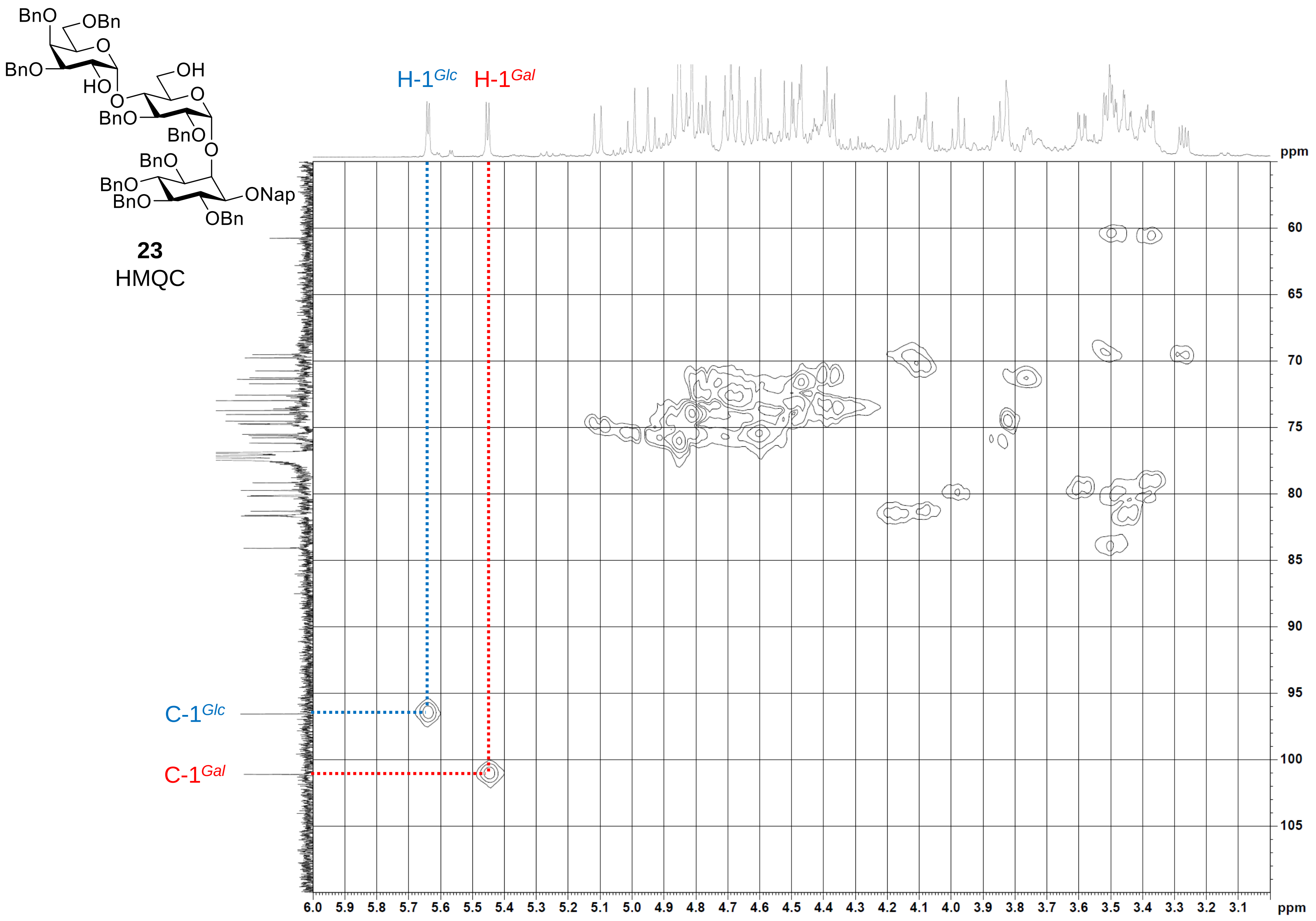
BnO
OBn
O
BnO
HO
OH
O
O
BnO
BnO
O
BnO
BnO
BnO
ONap
OBn
23
HMQC
H-1Glc
H-1Gal
C-1Glc
C-1Gal
ppm
60
65
70
75
80
85
90
95
100
105
6.0 5.9 5.8 5.7 5.6 5.5 5.4 5.3 5.2 5.1 5.0 4.9 4.8 4.7 4.6 4.5 4.4 4.3 4.2 4.1 4.0 3.9 3.8 3.7 3.6 3.5 3.4 3.3 3.2 3.1
ppm

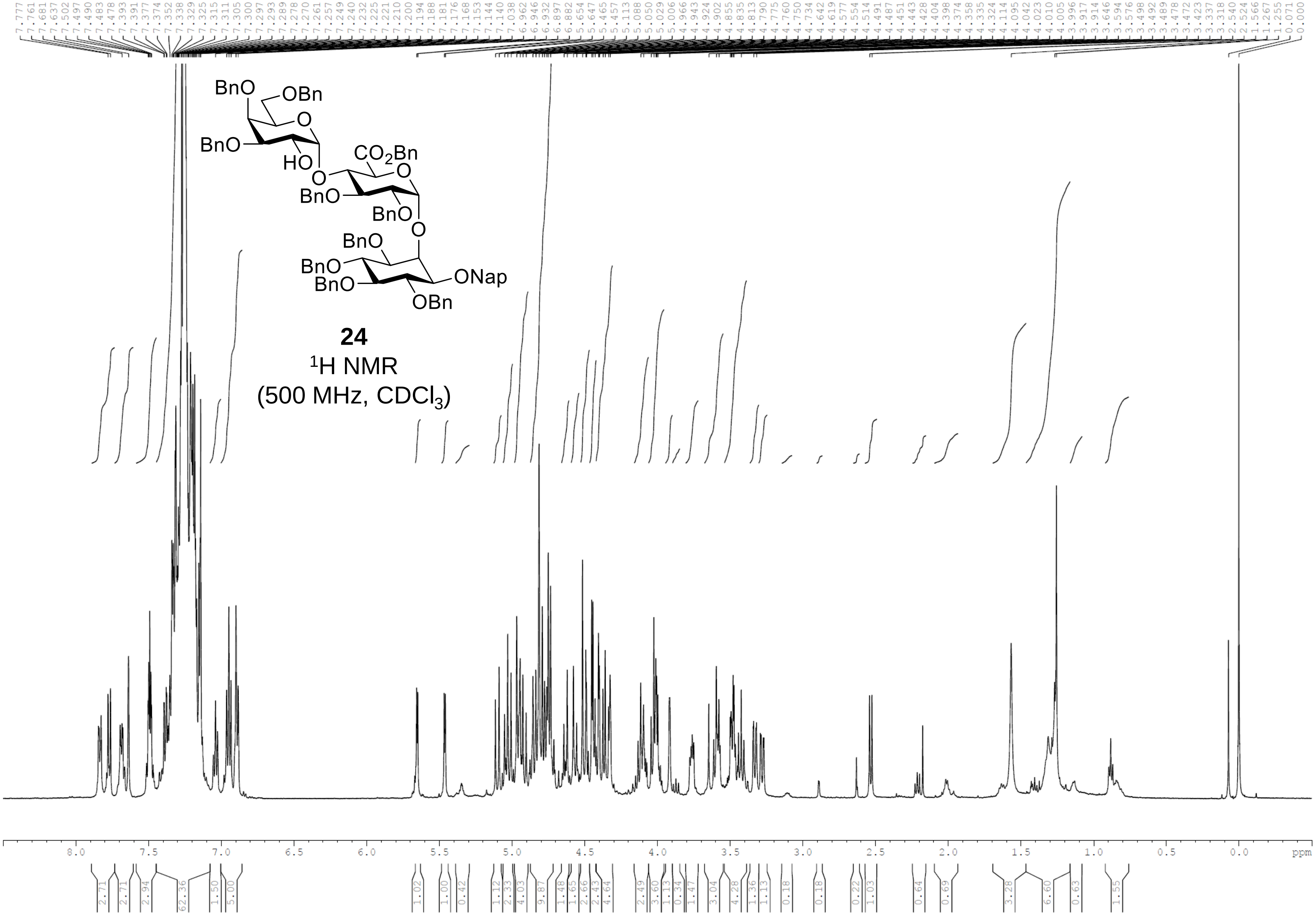

24
¹H NMR
(500 MHz, CDCl3)
BnO
OBn
O
BnO
HO
CO2Bn
BnO
ONap
OBn
ppm

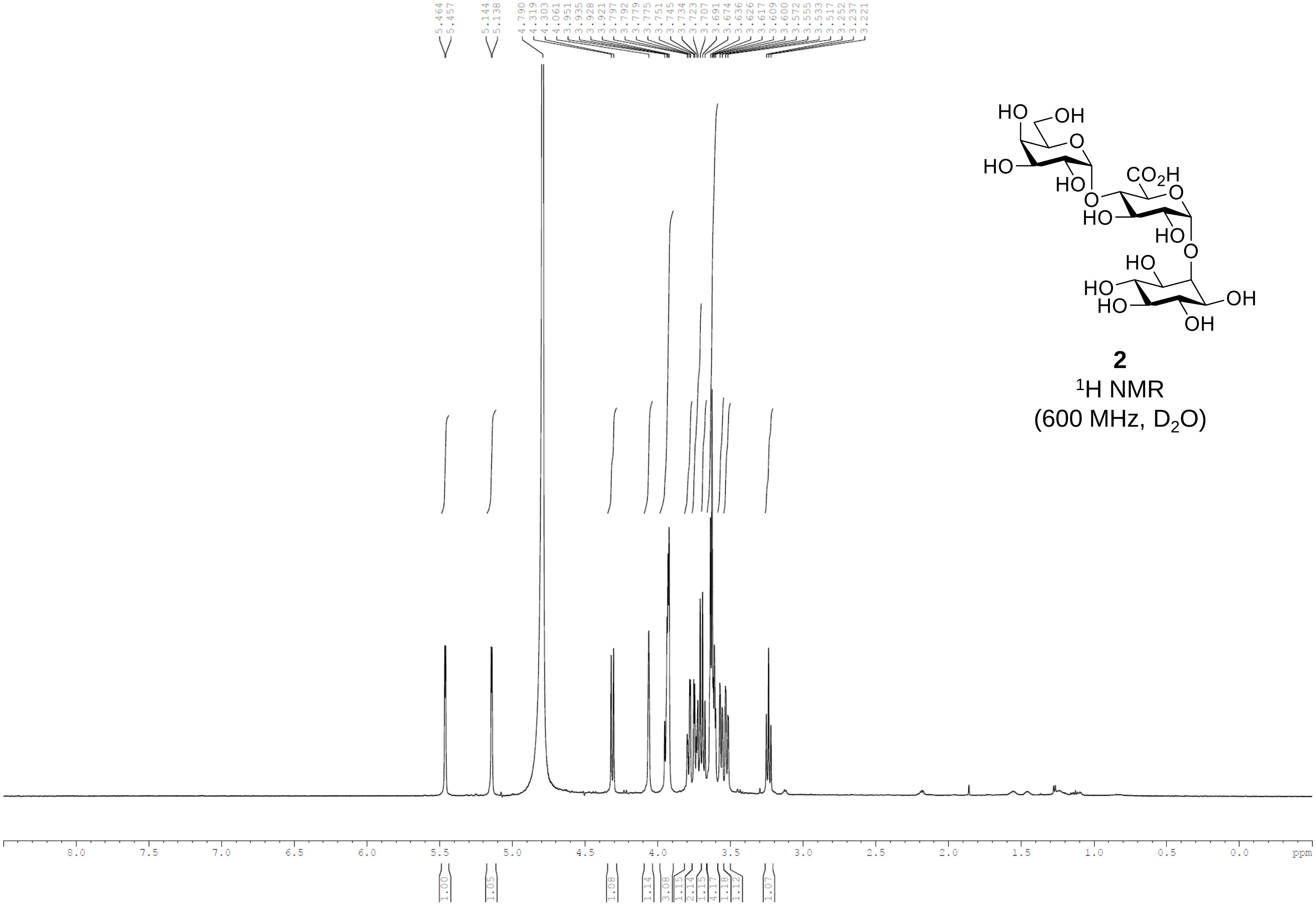

2

^{1}H NMR

(600 MHz, D_2O)

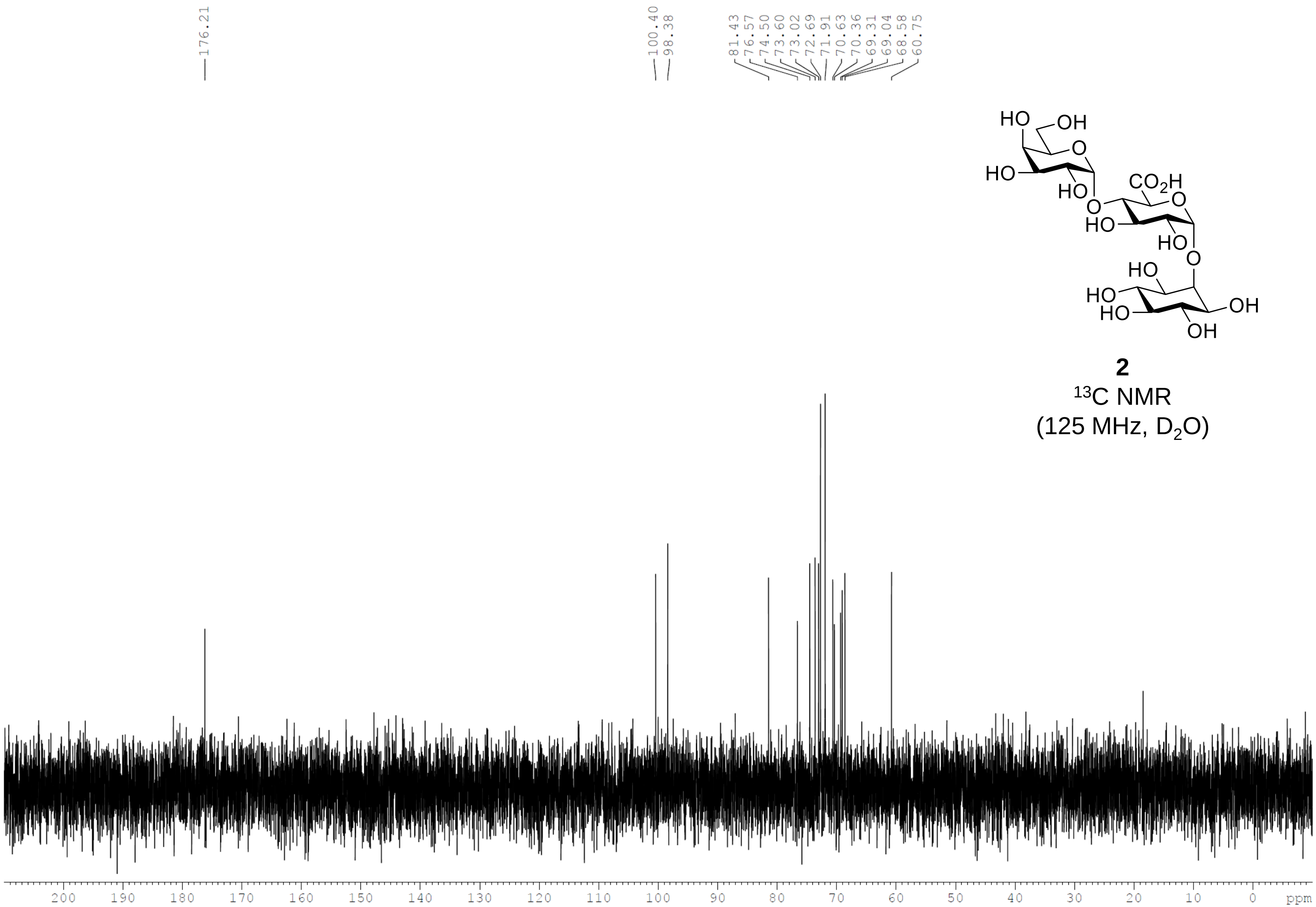

2

^{13}C NMR

(125 MHz, D_2O)

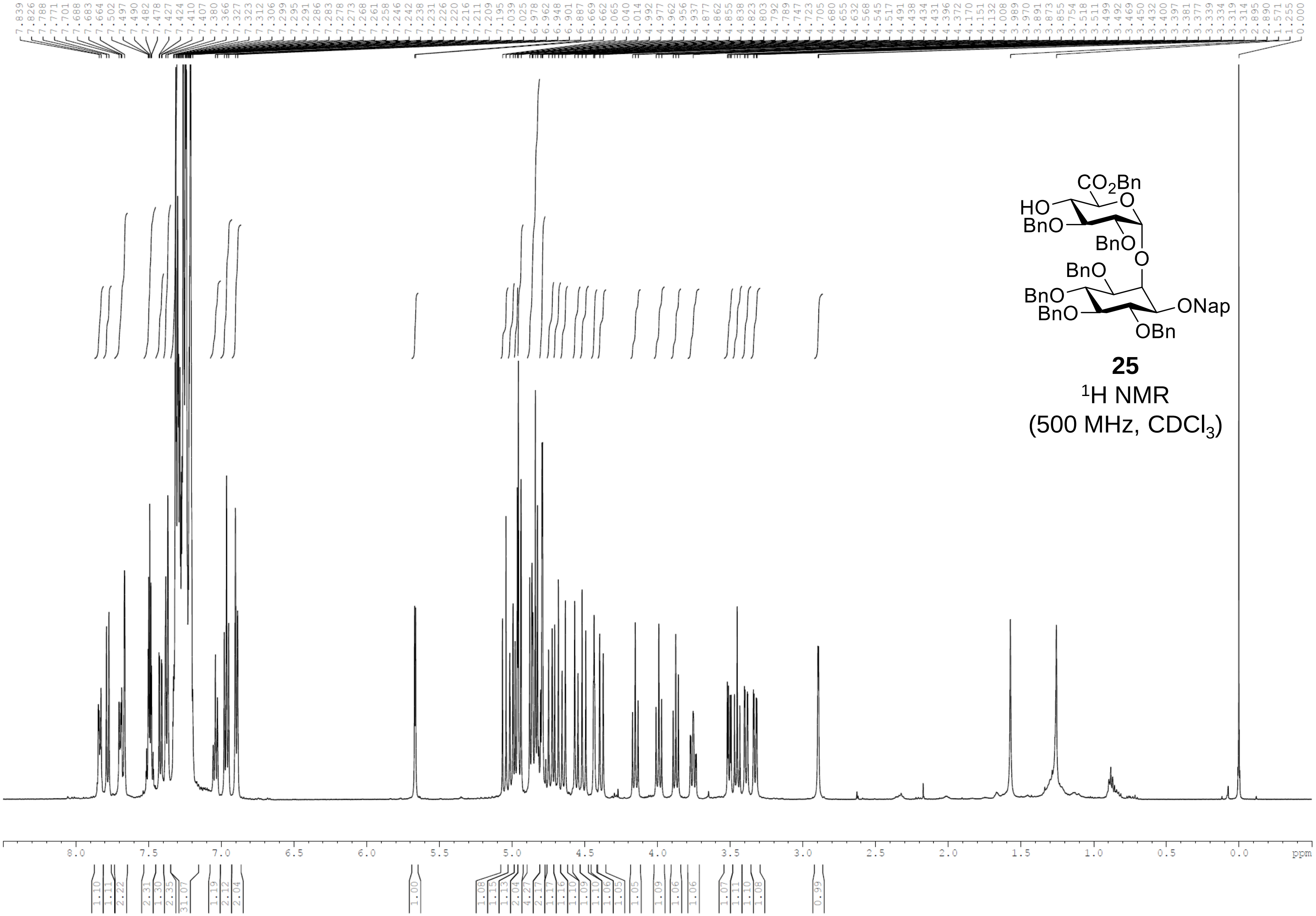

25

^{1}H NMR

(500 MHz, $CDCl_3$)

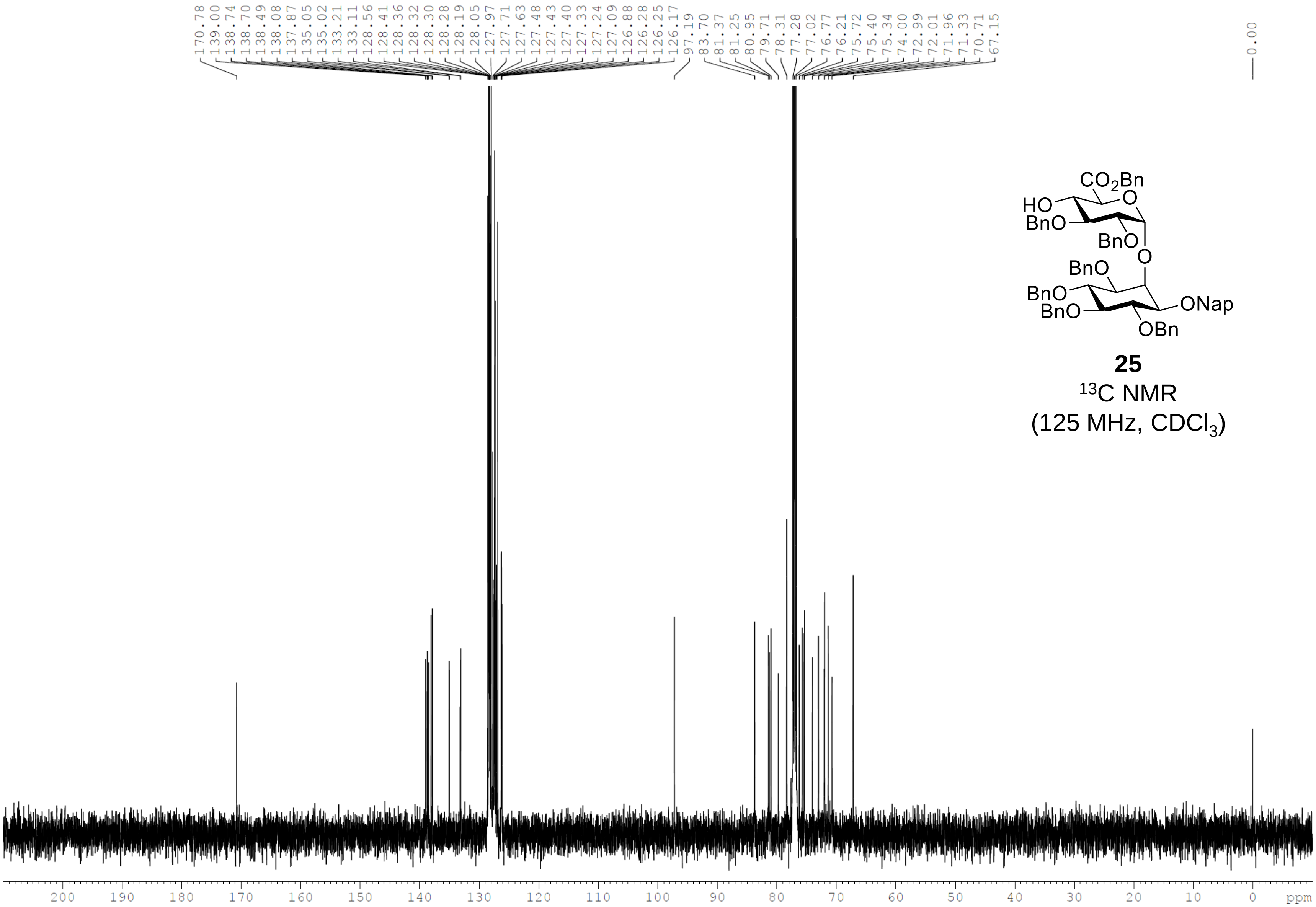

25

^{13}C NMR

(125 MHz, $CDCl_3$)

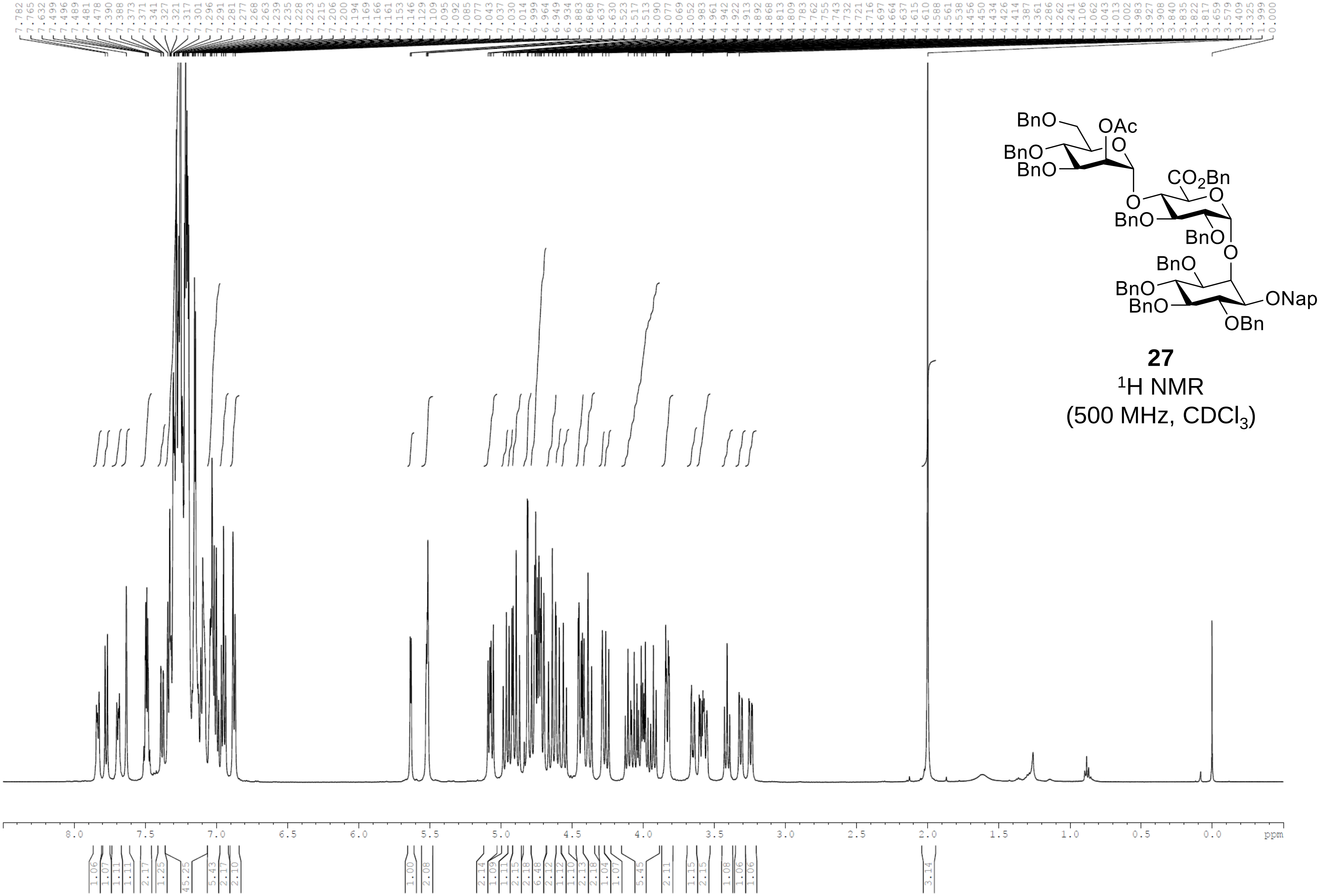

27

^{1}H NMR

(500 MHz, $CDCl_3$)

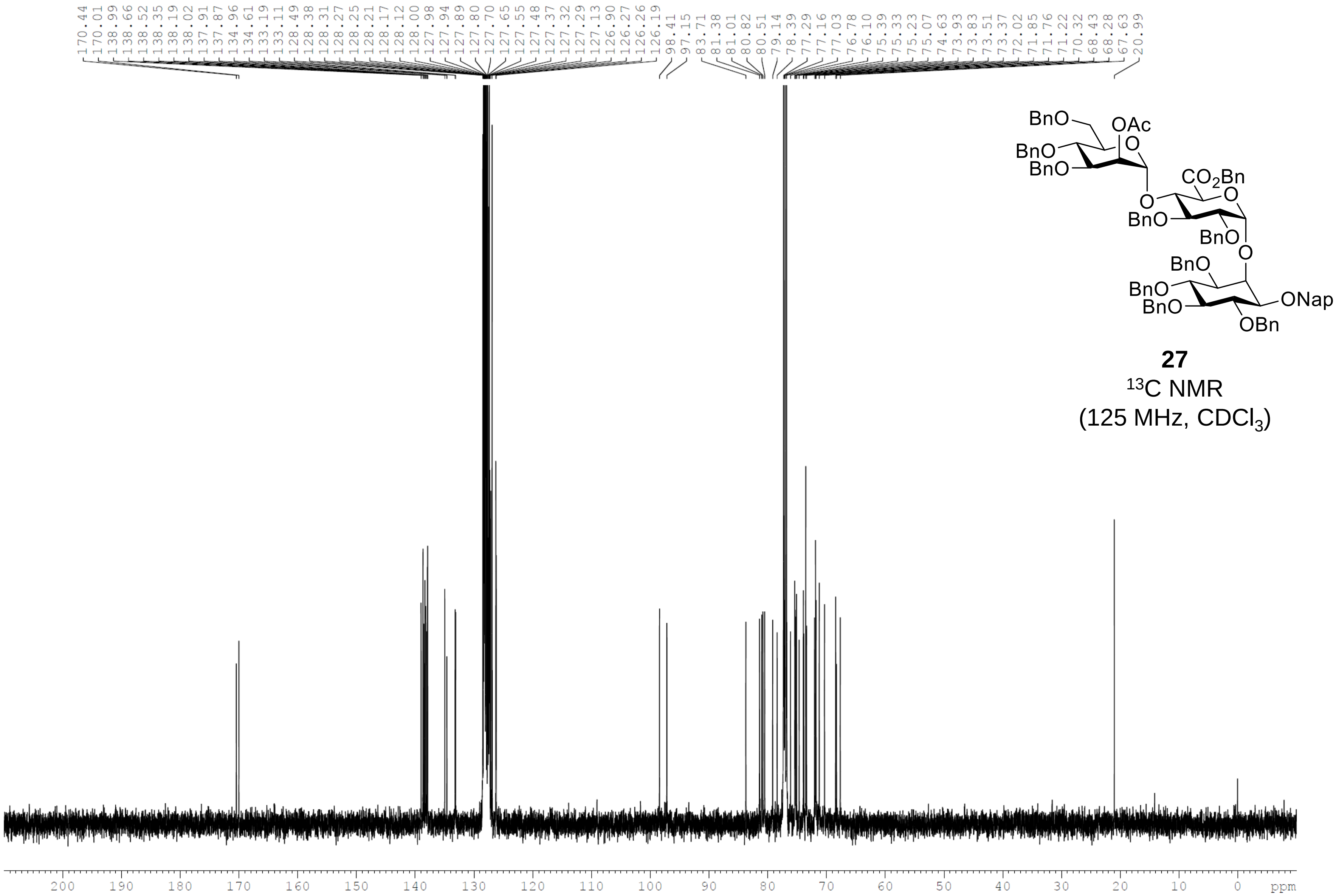
170.44
170.01
138.99
138.66
138.52
138.35
138.19
138.02
137.91
137.87
134.96
134.61
133.19
133.11
128.49
128.38
128.31
128.27
128.25
128.21
128.17
128.12
128.00
127.98
127.94
127.89
127.80
127.70
127.65
127.55
127.48
127.37
127.32
127.29
127.13
126.90
126.27
126.26
126.19
98.41
97.15
83.71
81.38
81.01
80.82
80.51
79.14
78.39
77.29
77.16
77.03
76.78
76.10
75.39
75.33
75.23
75.07
74.63
73.93
73.83
73.51
73.37
72.02
71.85
71.76
71.22
70.32
68.43
68.28
67.63
20.99
BnO
OAc
O
BnO
BnO
O
CO_2Bn
O
BnO
BnO
O
BnO
BnO
BnO
ONap
OBn
27
^{13}C NMR
(125 MHz, $CDCl_3$)
200
190
180
170
160
150
140
130
120
110
100
90
80
70
60
50
40
30
20
10
0
ppm

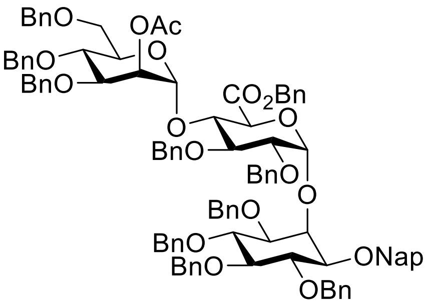
BnO
OAc
O
BnO
BnO
CO2Bn
O
O
BnO
BnO
O
BnO
BnO
BnO
ONap
OBn

27

HMQC

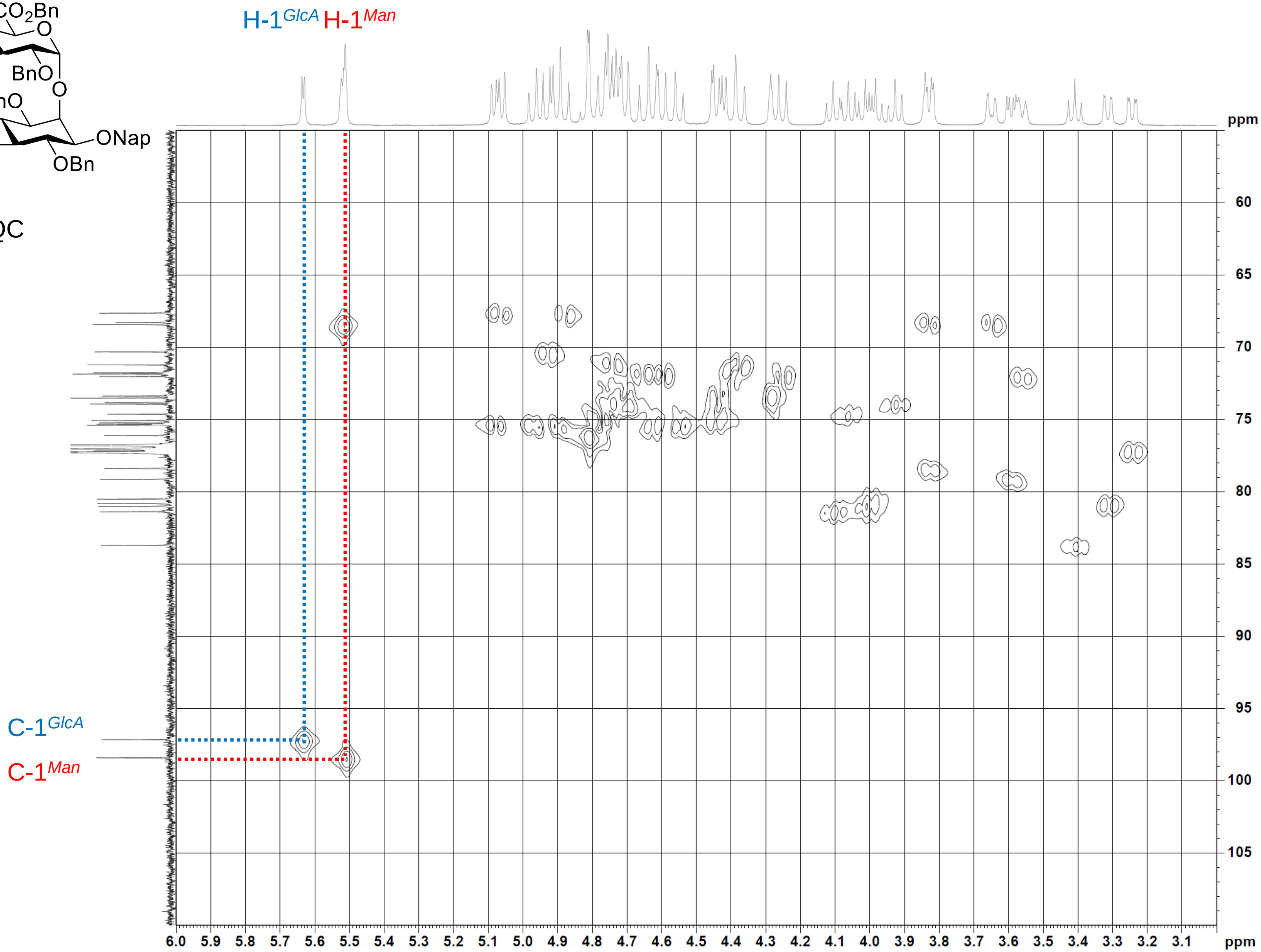
H-1GlcA
H-1Man
C-1GlcA
C-1Man
ppm
60
65
70
75
80
85
90
95
100
105
6.0 5.9 5.8 5.7 5.6 5.5 5.4 5.3 5.2 5.1 5.0 4.9 4.8 4.7 4.6 4.5 4.4 4.3 4.2 4.1 4.0 3.9 3.8 3.7 3.6 3.5 3.4 3.3 3.2 3.1
ppm

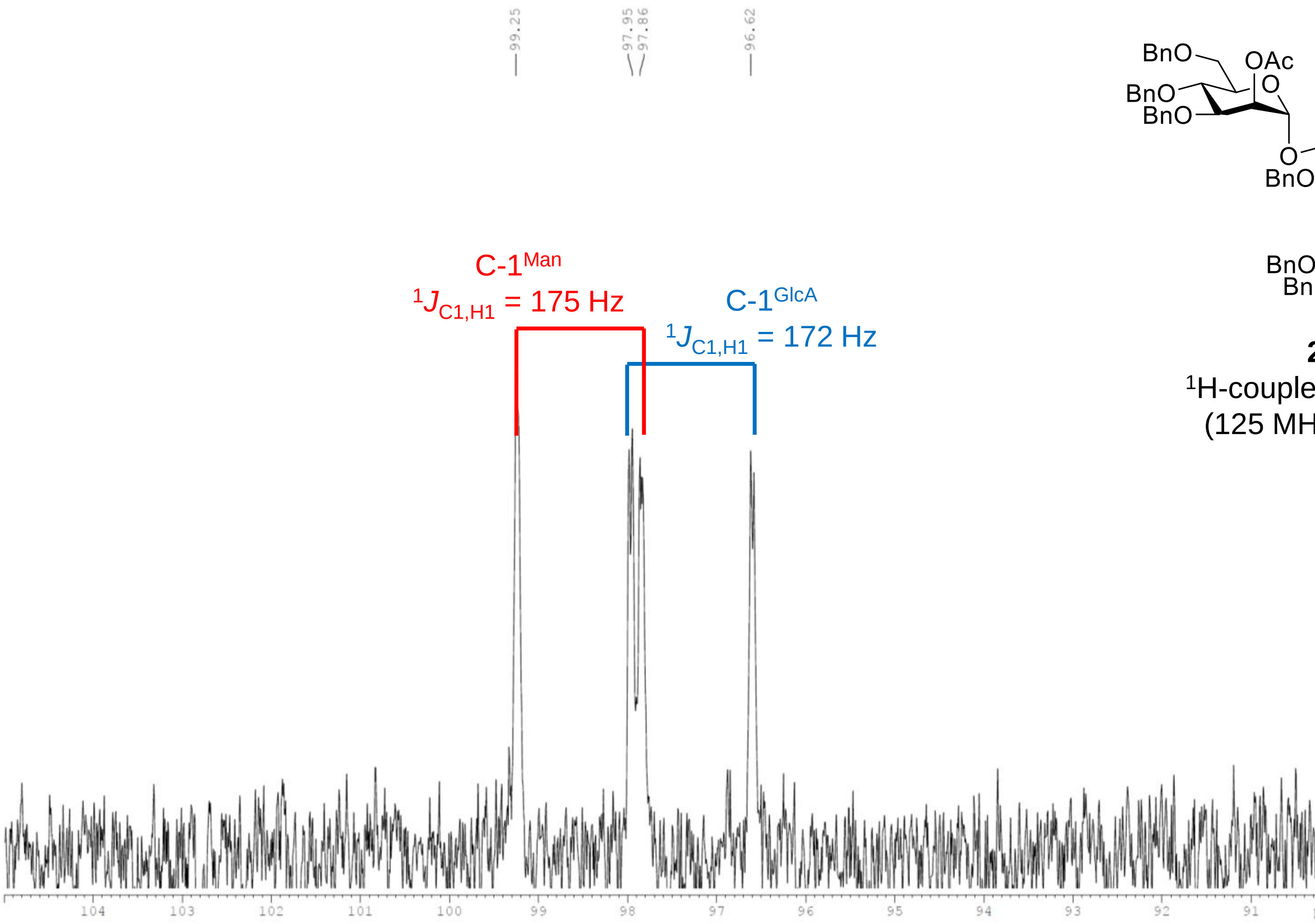

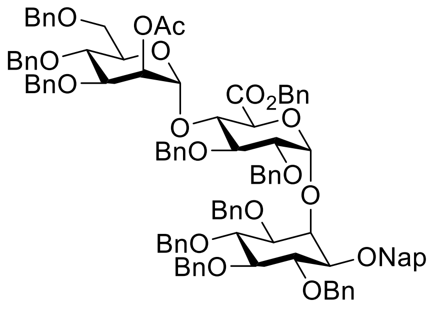

27

^1H-coupled ^{13}C NMR (125 MHz, $CDCl_3$)

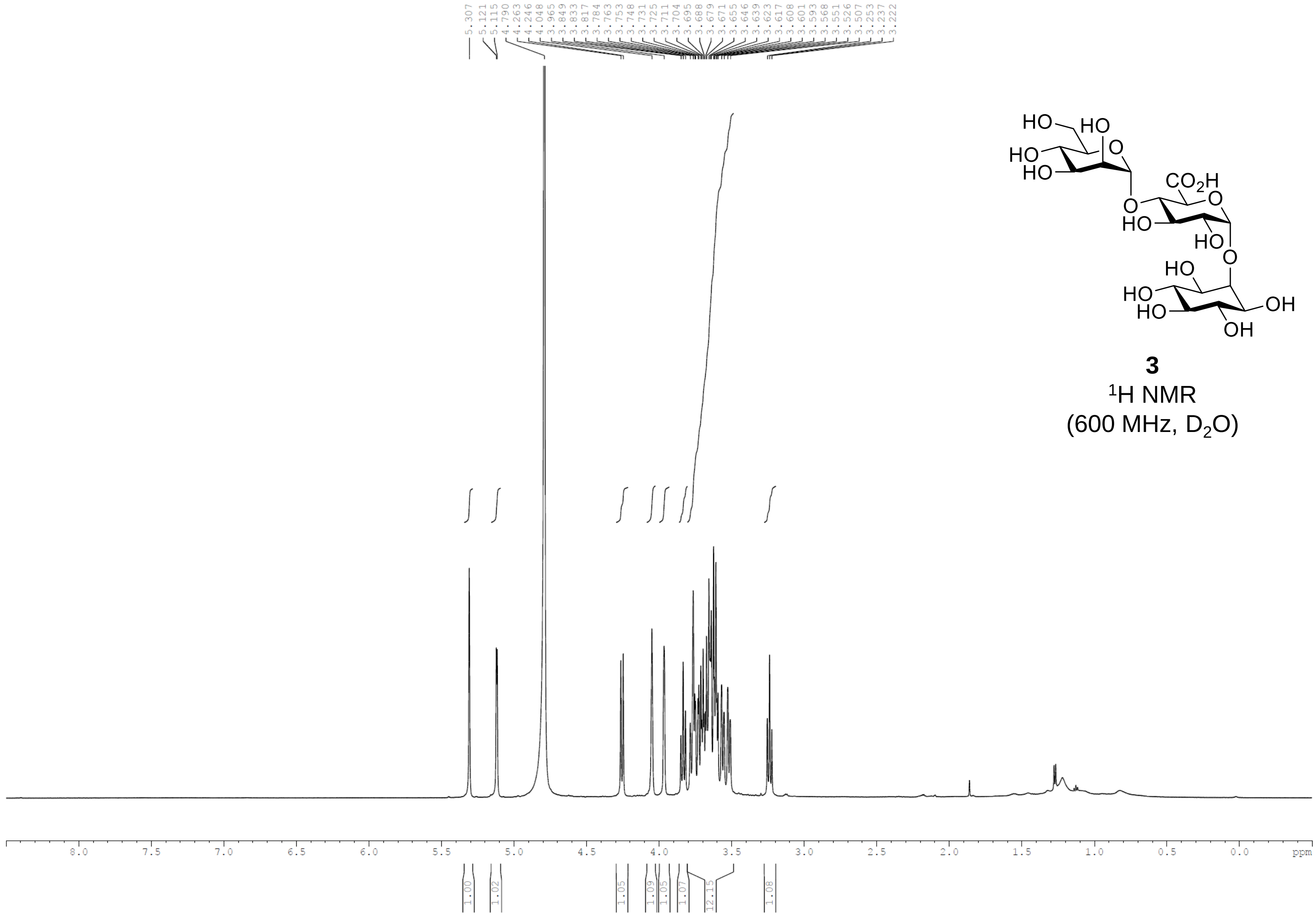

3

^{1}H NMR

(600 MHz, D_2O)

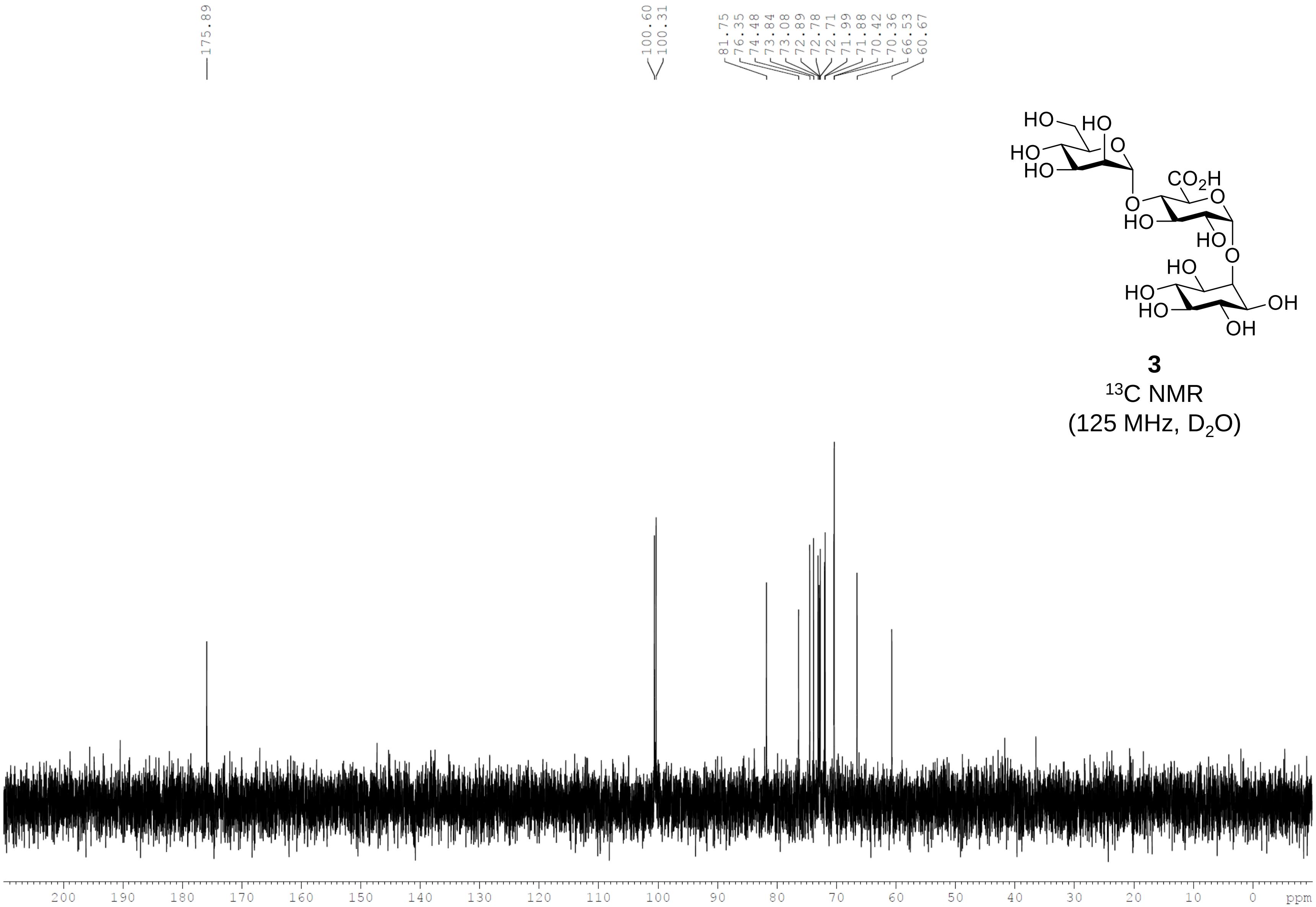

3

^{13}C NMR

(125 MHz, D_2O)
